# A cell-penetrating artificial metalloenzyme regulates a gene switch in a designer mammalian cell

**DOI:** 10.1038/s41467-018-04440-0

**Published:** 2018-05-16

**Authors:** Yasunori Okamoto, Ryosuke Kojima, Fabian Schwizer, Eline Bartolami, Tillmann Heinisch, Stefan Matile, Martin Fussenegger, Thomas R. Ward

**Affiliations:** 1 Department of Chemistry, University of Basel, Mattenstrasse 24a, BPR 1096, CH-4058 Basel, Switzerland; 2 Department of Biosystems Science and Engineering, ETH Zurich, Mattenstrasse 26, CH-4058 Basel, Switzerland; 3 Graduate School of Medicine, The University of Tokyo, 7-3-1 Hongo, Bunkyo-ku, Tokyo, 113-0033 Japan; 4 Department of Organic Chemistry, University of Geneva, CH-1211 Geneva, Switzerland

## Abstract

Complementing enzymes in their native environment with either homogeneous or heterogeneous catalysts is challenging due to the sea of functionalities present within a cell. To supplement these efforts, artificial metalloenzymes are drawing attention as they combine attractive features of both homogeneous catalysts and enzymes. Herein we show that such hybrid catalysts consisting of a metal cofactor, a cell-penetrating module, and a protein scaffold are taken up into HEK-293T cells where they catalyze the uncaging of a hormone. This bioorthogonal reaction causes the upregulation of a gene circuit, which in turn leads to the expression of a nanoluc-luciferase. Relying on the biotin–streptavidin technology, variation of the biotinylated ruthenium complex: the biotinylated cell-penetrating poly(disulfide) ratio can be combined with point mutations on streptavidin to optimize the catalytic uncaging of an allyl-carbamate-protected thyroid hormone triiodothyronine. These results demonstrate that artificial metalloenzymes offer highly modular tools to perform bioorthogonal catalysis in live HEK cells.

## Abstract (web summary)

Artificial enzymes can be used to elicit reactions in cells. Here, the authors developed such an artificial catalyst combined with a genetic switch, and showed that it was readily taken up by human cells and able to kick off a reaction cascade resulting in the biosynthesis of the desired product.

## Introduction

**Fig. 1:**
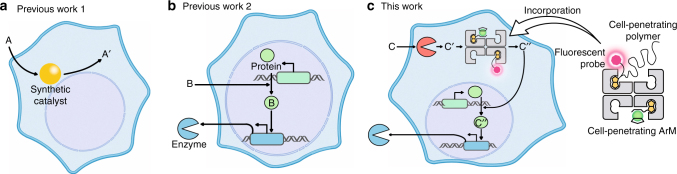
Prior art and concept of the work. **a** A synthetic catalyst (metal complex or nanoparticle) converts A to A′ intracellularly (A′ is either a fluorescent probe or a drug). **b** A designer mammalian cell engineered with a synthetic gene circuit responds to an external trigger molecule B. **c** Introduction of ArMs in designer cells for the control of a bioorthogonal reaction. The doubly caged molecule C is converted into C′ by an endogeneous natural enzyme. The bioactive molecule C″, which upregulates a synthetic gene circuit, is produced by the ArM. All figures presented in this study were created by authors

In recent years, there has been an increasing effort to exploit the cell as a test-tube to complement the biochemical reaction networks with abiotic reactions^1, 2^ (Fig. 1a). With this goal in mind, both organometallic complexes and nanoparticles have been shown to catalyze abiotic reactions in *Escherichia coli*^3–7^, mammalian cells^8–21^, and animals^22–25^. The outcome of such intracellular abiotic reactions has been mostly limited to a bioorthogonal output (i.e. uncaging of a fluorescent molecule or labeling of a protein) or a loss-of-function (i.e. drug-release that leads to cell death)^3–25^. In a limited number of cases however, a gain-of-function (productive modulation/activation of cellular function) by an intracellular abiotic reaction has been reported^26, 27^.

In addition to these efforts, metabolic engineering in *E. coli*, yeast, and higher organisms has received increasing attention thanks to its enormous potential to produce either high-added value products and biofuels or to cure diseases^28^. In this latter context, the assembly of gene switches allows for the construction of engineered mammalian cells that are capable of sensing the extracellular environment and producing output molecules on demand (Fig. 1b)^29–31^. Thus far, only genetically encodable modules have been integrated into designer mammalian cells. This limits the available intracellular reaction repertoire to those accessible from engineered natural enzymes.

**Fig. 2:**
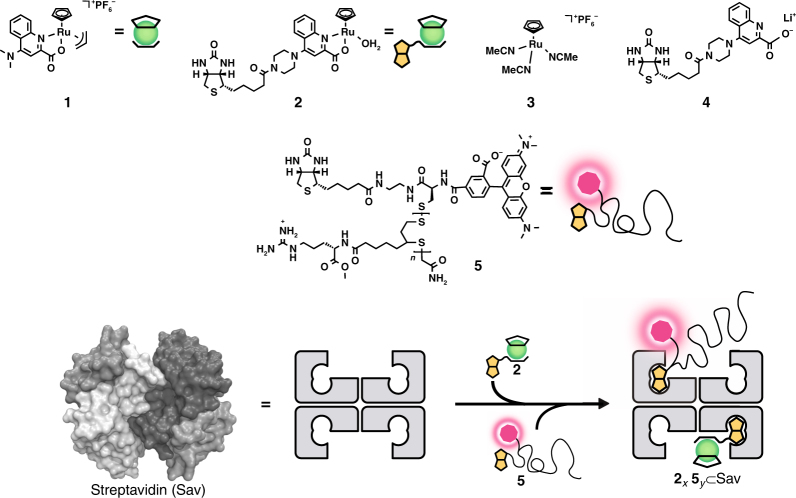
Assembly of cell-penetrating ArMs. Ruthenium complexes **1** and **2** catalyze a bioorthogonal uncaging reaction. The biotinylated cell-penetrating poly(disulfide) (CPD) bears a fluorescent TAMRA moiety **5** allowing the monitoring of cellular uptake. Incorporation of both biotinylated moieties **2** and **5** in various ratios (*x* and *y*) in tetrameric Sav affords a cell-permeable ArM for the uncaging of allyl carbamate-containing substrates within cells

Artificial metalloenzymes (ArMs hereafter), which result from incorporation of an organometallic moiety within a protein scaffold, combine attractive features of both homogeneous- and enzymatic catalysts (Fig. 1c and Fig. 2)^32, 33^. Most recently, the groups of Tezcan^34^ and Ward^6^ have demonstrated the possibility to assemble and use ArMs in vivo in the periplasm of *E. coli*. As a next step, we aim to integrate an ArM in a designed mammalian cell to upregulate the expression of a reporter protein via a complex reaction cascade (Fig. 1c). To achieve this goal, the following challenges need to be addressed: (i) the efficient uptake of an ArM into mammalian cells; (ii) the assembly of a gene switch that senses and responds to the product of the ArM resulting in (iii) the intracellular upregulation of the gene switch by the ArM.

Herein we demonstrate the proof-of-principle of an intracellular abiotic reaction enabled by an ArM modified with a cell-penetrating module. This cell-penetrating ArM is taken up into mammalian cells and catalyzes an abiotic reaction, leading to the upregulation of a designed gene circuit.

## Results

### Design of a cell-penetrating artificial metalloenzyme

Thus far, most of the reported intracellular abiotic catalysis has relied on the catalysts’ inherent cell permeability: only few reports have used cell-permeable carrier modules^13, 16, 19, 20^. In the absence of a cell-permeable moiety, the efficiency of the catalyst’s cellular uptake has been a matter of controversy^14, 35^. To achieve efficient delivery of an organometallic catalyst into cells, we capitalize on the homotetrameric nature of a streptavidin scaffold to combine an abiotic biotinylated catalyst with a biotinylated cell-penetrating moiety. Additional attractive features of ArMs based on the biotin–streptavidin technology include: (i) the possibility to optimize the catalytic performance using genetic means^6, 36^ and (ii) protection of the precious metal cofactor against detrimental cellular components^6, 37^. We hypothesized that this Sav-based approach to assemble a cell-permeable ArMs may provide a versatile tool for the introduction of synthetic catalysts into cells.

With the aim of combining a gene switch with an abiotic reaction catalyzed by an ArM in a designer mammalian cell, we combined a ruthenium catalyst for the intracellular *O*-allyl carbamate cleavage^12, 14, 19, 35^ with a gene switch that is upregulated in the presence of the thyroid hormone, triiodothyronine (T_3_)^38^. The T_3_ hormone is known to affect thermogenesis, carbohydrate metabolism, and lipid homeostasis in all tissues. Our T_3_-responsive gene switch has been shown to work in various cell lines, including HEK-293T, Hela, immortalized human mesenchymal stem cells (hMSC-TERT), HT-1080, and CHO-K1 cells^38^. Among these cell lines, we selected HEK-293T cells, which is the most responsive. Building on the ruthenium complex **1** introduced by Meggers and co-workers^14^, the biotinylated ruthenium complex **2** was prepared in situ by mixing [CpRu(NCCH_3_)_3_](PF_6_) **3** and the biotinylated ligand **4** in a 1:1 ratio. (Fig. 2). We selected the cell-penetrating poly(disulfide) (CPD) **5**, which was previously developed by us, as a cell-permeable module (Fig. 2)^39, 40^. CPD is taken up via dynamic covalent disulfide exchange with thiols on mammalian cell surfaces. The presence of glutathione in the cytosol leads to depolymerization of the CPD, thus alleviating continual membrane-perturbing activities and reducing cytotoxicity compared to traditional arginine-rich cell-penetrating peptides^40, 41^. Relying on the versatility of the CPD as demonstrated for Hela cells^39^ and Drosophila S2 cells^40^, we hypothesized that these would be applicable for HEK-293T cells as well.

### In vitro optimization of the ArM

**Fig. 3:**
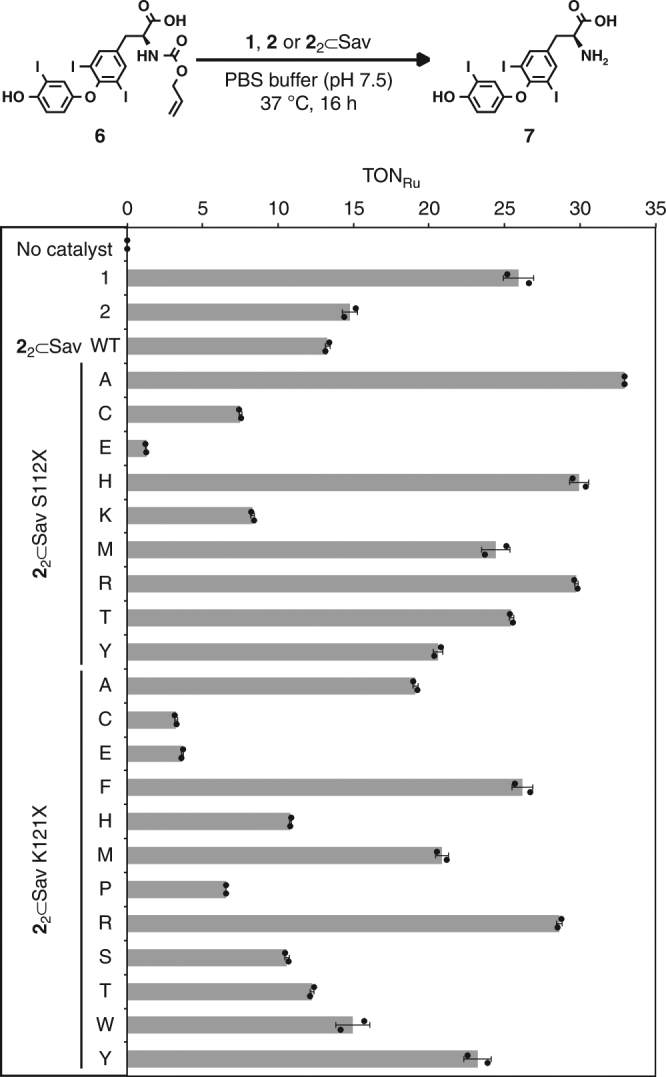
In vitro genetic optimization of the ArM. The ruthenium-catalyzed uncaging of AT_3_ **6** affords the thyroid hormone triiodothyronine T_3_ **7**. Turnover numbers (TON) are based on yields of T_3_ **7** determined by UPLC with an internal standard. Experiments were performed at 37 °C for 16 h; **1**, **2** (1 μM) or **2**_2_ ⊂ Sav (0.5 μM), AT_3_ **6** (100 μM) in PBS (pH 7.5). Data are the means ± standard deviation of duplicate reactions

To ensure efficient coupling between the reaction catalyzed by the ArM and the gene switch, the performance of the ArM **2**_2_ ⊂ Sav (the subscript indicates the number of biotinylated catalyst moieties **2** added to the homotetrameric Sav) was optimized by single point mutations of the Sav scaffold for the *O*-allyl carbamate cleavage of the caged hormone (AT_3_) **6**, to yield T_3_ **7** (Fig. 3, Supplementary Figure 1). The ruthenium complex **1** (ref.^14^) displayed higher activity than both the biotinylated ruthenium complex **2** and the corresponding ArM **2**_2_ ⊂ Sav wild type in vitro. Genetic optimization of the catalytic performance of **2**_2_ ⊂ Sav was achieved by site-directed mutagenesis at position Sav S112X or K121X, which are the putative closest lying amino acid residues^36^. The mutant **2**_2_ ⊂ Sav S112A had a 2.5-fold higher TON (turnover number) than the wild-type ArM and a comparable activity to the catalyst **1**. None of the screened double mutants outperformed **2**_2_ ⊂ Sav S112A, which was selected for subsequent studies.

### Designer cells with a T_3_-gene switch

**Fig. 4:**
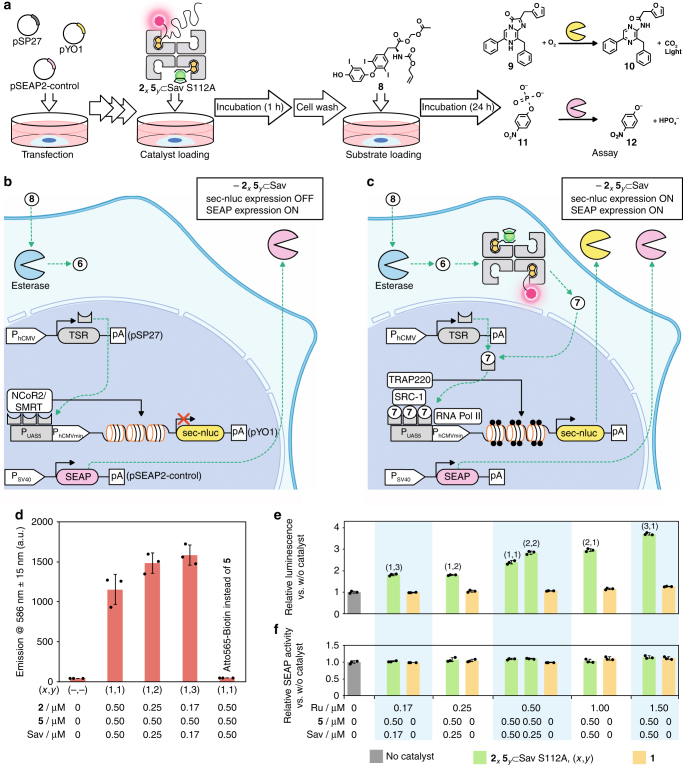
An ArM-catalyzed intracellular reaction induces a gene switch. **a** Following seeding and incubation (43 h), the HEK-293T cells (approx. 5 × 10^6^ cells/10 cm dishes) were transfected with pSP27 (P_hCMV_-TSR-pA), pYO (P_UAS5_-sec-nluc-pA), and pSEAP2-control (P_SV40_-SEAP-pA). The resulting transfected cells were dispensed into a collagen-coated 24-well cell culture plate with two times diluted concentration and incubated overnight. Cells were treated with either the ruthenium complex **1** or the ArM **2**_*x*_**5**_*y*_ ⊂ Sav S112A (0.17–1.5 μM ruthenium). After 1 h, the cells were washed and subsequently incubated with AM-AT_3_ **8** (4 μM, 24 h). Activities of the sec-nluc (luminescent conversion of **9** into **10**) and SEAP (conversion of **11** into chromophore **12**) were quantified using the supernatant of the cell culture medium. **b** A designer cell in the absence of ArM **2**_*x*_**5**_*y*_ ⊂ Sav S112A. In the absence of T_3_ **7**, TSR associates with corepressors (SMRT/NcoR2) on the P_UAS5_ and deacetylation of histone is triggered thus inhibiting gene expression. **c** The designer cell including the ArM **2**_*x*_**5**_*y*_ ⊂ Sav S112A. AM-AT_3_ **8** is sequentially converted into AT_3_ **6** and T_3_ **7** by endogenous esterases and the ArM respectively. TSR bound to T_3_ **7** interacts with coactivators (SRC-1, TRAP 220) on the P_UAS5_ to trigger histone acetylation, resulting in gene expression. In both **b** and **c**, SEAP is continuously expressed: its phosphatase activity against *p-*nitrophenylphosphate **11** allows to estimate the cell viability. **d** The uptake of ArMs revealed by the fluorescence from either the biotinylated TAMRA- or the Atto565-moieties. After 24 h incubation with AM-AT_3_ **8**, cells were analyzed by flow cytometry (ex. 561 nm, em. 586 ± 15 nm band-pass). As a control experiment, Atto565-biotin was used instead of CPD **5**. Quantification of the enzymatic activities of **e** the expressed sec-nluc induced by the uncaging activity of ArM **2**_*x*_**5**_*y*_ ⊂ Sav S112A (green), the Ru-complex **1** (beige), or without a catalyst (gray) and **f** the constitutively expressed SEAP after 24 h. The ratio of biotinylated catalyst **2** and CPD **5** added to the tetrameric Sav scaffold is described as (*x*, *y*). Data displayed are the means ± standard deviation of experiments performed in triplicate

In order to monitor the output of the reaction cascade, which is reflected by the transgene expression level resulting from the T_3_-gene switch, we slightly adapted a previously reported thyroid hormone-responsive gene switch in HEK-293T cells (Fig. 4a–c)^38^. This modified thyroid hormone-responsive gene switch consists of a set of two plasmids, pSP27 and pYO1. The plasmid pSP27 codes for a synthetic thyroid hormone receptor (TSR), which is a fusion protein comprised of the DNA-binding domain of yeast Gal4 and the T_3_-binding domain of human thyroid receptor α (P_hCMV_-TSR-pA) (P_hCMV_: human cytomegalovirus promoter). The plasmid pYO1 comprises a Gal4-specific operator sequence (P_UAS5_) connected to a minimal hCMV promoter (P_hCMVmin_) that drives expression of a secreted nanoluc (sec-nluc), a potent bioluminescence reporter (P_UAS5_-sec-nluc-pA). In the absence of T_3_ **7**, TSR binds to P_UAS5_ and recruits endogenous corepressors, silencing mediators for retinoid or thyroid hormone receptors (SMRT)/nuclear receptor corepressor 2 (NcoR2), leading to histone deacetylation. As a result, the expression of sec-nluc is repressed (Fig. 4b). In the presence of T_3_ **7**, the TSR interaction with T_3_ recruits coactivators: steroid receptor coactivator 1 (SRC-1) and a 220-kDa thyroid hormone receptor-associated protein complex component (TRAP 220). These coactivators trigger histone acetylation to initiate sec-nluc expression, which is monitored by the luminescent conversion of furimazine **9** into product **10** (Fig. 4a). As an indicator of the cell viability, a human placental secreted alkaline phosphatase (SEAP), was co-expressed constitutively by pSEAP2-control (P_SV40_-SEAP-pA) (P_SV40_: similian virus 40 promoter). Hydrolysis of *p-*nitrophenylphosphate **11** affords *p-*nitrophenolate **12**, which can be monitored spectrophotometrically at 405 nm (Fig. 4a–c).

Transport of native T_3_ **7** into cells usually relies on a transporter-mediated process^42^. As it was not clear whether the AT_3_ **6** is transported into cell via this pathway, we modified AT_3_ **6** to increase its cell permeability. For this purpose, we esterified the carboxylic group of AT_3_ **6** with an acetoxymethyl (AM) group to yield AM-AT_3_ **8** (refs.^43, 44^). Upon passive diffusion of AM-AT_3_ **8** into the cytosol, endogenous esterases rapidly hydrolyze it to produce the allyl-carbamate protected AT_3_ **6** (Fig. 4b, c).

First, we investigated the dose-dependence of T_3_ **7** and AM-AT_3_ **8** against the T_3_-responsive gene switch (pSP27 and pYO1) and the pSEAP-2 control (Supplementary Figure 2). When using T_3_ **7**, the luminescence intensity derived from the expressed sec-nluc activity reached a plateau at very high gene expression level (i.e. >6 nM T_3_ **7**). At concentrations below 500 nM, AM-AT_3_ **8** did not induce sec-nluc expression. This highlights the efficient caging effect of the allyl carbamate moiety of AM-AT_3_ **8**. Gratifyingly, the phosphatase activity of SEAP did not decrease in the presence of AM-AT_3_ **8**: we thus conclude that it does not exhibit cytotoxicity at these concentrations.

### Intracellular abiotic reaction

After the in vitro identification of the most active ArM for the allyl-carbamate cleavage of AT_3_ **6** (see Fig. 3), we next assembled a cell-penetrating ArM **2**_*x*_**5**_*y*_ ⊂ Sav S112A by sequential incorporation of the ruthenium complex **2** and biotinylated CPD **5** into Sav S112A (Fig. 2, Supplementary Figure 3 and Supplementary Discussion). Capitalizing on the four biotin-binding sites present in Sav, the ratio of the ruthenium catalyst **2** and CPD **5** was optimized. Based on a previous report^40^ and a preliminary screening experiment (Supplementary Figure 4), the concentrations of CPD **5** and AM-AT_3_ **8** were set to 0.5 and 4 μM, respectively. These concentrations were selected to maximize the cellular uptake of the ArM with the minimum cell toxicity and minimize the background luminescence caused by AM-AT_3_ **8**.

The designer HEK-293T cells transfected with the T_3_-responsive gene switch were treated with ArM **2**_*x*_**5**_*y*_ ⊂ Sav S112A. Following incubation and careful washing of the cells to remove surface-adsorbed ArM, the substrate AM-AT_3_ **7** (4 μM) was added (Fig. 4a). We monitored the ArM’s uptake in the designer cell by the confocal microscopy (Supplementary Figure 5) and flow cytometry (Fig. 4d) before and after the reaction respectively. For comparison, the CPD **5** was replaced by Atto565-Biotin, which exhibits similar spectroscopic features to CPD **5** but is not internalized by covalent-mediated translocation through the cell-membrane (Supplementary Figure 6 and Supplementary Discussion), to afford **2**_1_(Atto565-Biotin)_1_ ⊂ Sav. In the case of **2**_1_(Atto565-Biotin)_1_ ⊂ Sav, the fluorescence derived from Atto565-Biotin was not detectable either by confocal microscopy or flow cytometry. This indicates that the ArM devoid of CPD **5** is not cell-permeable. In contrast, the cellular uptake of ArMs **2**_*x*_**5**_*y*_ ⊂ Sav was confirmed by the pronounced increase in fluorescence within the cells caused by the presence of TAMRA moieties on the ArMs.

As for the intracellular reactions, the luminescence intensities resulting from the induced sec-nluc activity were markedly higher in the presence of the ArM **2**_1_**5**_1_ ⊂ Sav S112A than with model catalyst **1** (Fig. 4e). Although its activity in vitro is comparable to that of the ArM **2**_2_ ⊂ Sav S112A (Fig. 3), the use of complex **1** did not lead to higher luminescence intensities than when the designer cells were treated with the substrate AM-AT_3_ **8** but in the absence of any Ru-catalyst (Fig. 4e). The cofactor **2** alone and the ArM **2**_1_ ⊂ Sav S112A were also examined and found not to turn on the gene switch (Supplementary Figure 7 and Supplementary Discussion). This corroborates the flow cytometry observations, highlighting the limited uptake in HEK-293T cells when low Ru-concentrations are used (e.g. <1.5 μM)^35^. These results confirm that only cell-penetrating ArMs turn on the T_3_-responsive gene switch by producing T_3_ **7** via the intracellular uncaging of AT_3_ **6**. Increasing the Ru-catalyst **2** to CPD **5** ratio led to higher luminescence intensities: the highest activity was observed with the ArM **2**_3_**5**_1_ ⊂ Sav S112A. This highlights the importance of the “membrane permeability” and the “internalization” features of the cell-penetrating ArM. The cell viability, reflected by the SEAP activity, is essentially constant for all conditions tested. This confirms that there is no critical cytotoxicity for the intracellular reaction cascade (Fig. 4f).

## Discussion

Herein, we have demonstrated the versatility of a complex intracellular reaction cascade that results from combining a cell-penetrating ArM with a synthetic gene circuit. The uncaging reaction catalyzed by the ArM leads to the exquisite control of a designer cell, ultimately upregulating the activity of a genetically encoded sec-nluc. The observed output of this system is unambiguous. However,  further improvement would be desirable for practical use. For this purpose, in vivo directed evolution of ArMs have proven versatile^6^. Also, it may be possible to stably integrate the gene switch into designer cells to allow for the selection of the best performing single cell clone^45^.

Although cell-targeting modules have previously been combined with organometallic moieties to catalyze intracellular transformations^19^, the use of a Sav-based ArM combines several attractive features including: (i) a straightforward means to assemble and optimize the ArMs by chemical and genetic strategies, (ii) easy modulation of the catalyst:CPD ratio to fine-tune the uptake and the activity of the ArM, (iii) a means to visualize by fluorescence the internalization of the ArM, and (iv) the shielding of the precious metal moiety within a protein scaffold^37^.

This proof-of-concept highlights the gain of cellular function that critically relies on a reaction requiring components that are not genetically encoded. This strategy may be extended to new-to-nature reactions based on related ArMs: Suzuki-coupling, metathesis and C–H activation^33^. This may allow to endow mammalian cells with highly versatile catalytic function. We thus believe that this study may offer a novel means to engineer cellular function via highly modular cell-penetrating ArMs.

## Methods

### Synthesis of chemical compounds

Details are collected in the Supplementary Information (Supplementary Figures 8–17 and Supplementary Methods)

### Stock solutions of Sav, **1**, **2**, ArM **2**_*x*_ ⊂ Sav, and ArM **2**_*x*_**5**_*y*_ ⊂ Sav

Sav-stock solutions (stock **A**) were prepared in phosphate-buffered saline (PBS) (pH 7.5) or water to yield 800 μM free biotin-binding site (referred to as FBBS). A stock solution (stock **B**) of the ruthenium complex **1** (2 mM) was prepared in dimethyl sulfoxide (DMSO). A stock solution (stock **C**) of the ruthenium complex **2** (5 mM) was prepared by mixing [CpRu(NCCH_3_)_3_](PF_6_) **3** (10 mM in DMF) and the biotinylated ligand **4** (Supplementary Figure 9) (10 mM in DMF) in a 1:1 ratio. These stock solutions were freshly prepared for each experiment. The ArM **2**_*x*_  ⊂  Sav was prepared using the conditions listed in Supplementary Tables 1–3, respectively. Stock **C** was incubated with water for 10 min prior to the addition of stock **A**. The ArM **2**_*x*_**5**_*y*_ ⊂ Sav was prepared using the conditions collected in Supplementary Tables 4–9. After mixing the ArM **2**_*x* _ ⊂  Sav and water, CPD **5** was added and incubated for 2 h.

### **Screening Sav for the uncaging of AT**_**3**_**6**

Prior to Sav mutant screening, potassium isocyanoacetate was tested as an irreversible inhibitor for the catalytic activity of the ruthenium complexes **1**, **2** and ArM **2**_2_ ⊂ Sav (see Fig. 3 and Supplementary Figure 1). Allyl carbamate caged amino-coumarin **13** (see Supplementary Figure 18a) was used as a substrate. The reaction conditions are collected in Supplementary Table 10. Experiments were performed at 37 °C in a 96-well plate. The fluorescence intensity at 450 nm derived from the produced 7-amino-4-methylcoumarin **14** (excitation at 375 nm) was monitored with a Tecan Infinite M1000 Pro (Supplementary Figure 18b). Potassium isocyanoacetate was found to efficiently inhibit the catalytic activity of ruthenium complexes **1**, **2** and ArM **2**_2_ ⊂ Sav. Based on this, potassium isocyanoacetate was added to the reaction mixture to quench the reaction during the genetic optimization of the ArM’s activity. For the screening of Sav variants, AT_3_ **6** was used as a substrate. Reaction conditions are listed in Supplementary Table 11 and were performed at 37 °C in an HPLC vial. Potassium isocyanoacetate (500mM in water, 4 μL), tryptophanamide hydrochloride (10mM in water, 20μL) as an internal standard, acetonitrile (800μL), and PBS (400 μL) were added to the reaction mixture. The resulting solution was centrifuged (10,000*g*) and analyzed by UPLC-MS (Supplementary Figure 19). TONs were determined based on the calibration curve of T_3_ **7** (Supplementary Figure 20).

### Cell culture and transfection

The following three-cell culture media were prepared: medium A [Dulbecco’s modified eagle medium (DMEM; Invitrogen) containing 10% (v/v) fetal bovine serum (FBS; Sigma-Aldrich), 1% (v/v) penicillin/streptomycin (PS) solution (Sigma-Aldrich)], medium B [DMEM containing 1% (v/v) PS], medium C [DMEM containing 5% (v/v) charcoal stripped FBS (Gibco), 1% (v/v) PS]

Daily cell culture: HEK-293T cells (DSMZ: ACC-635) were cultivated in medium A at 37 °C in a humidified atmosphere containing 5% CO_2_. For serial passage of these cells, 0.05% Trypsin-EDTA (Gibco) was used. For HEK-293T cells which we used in this paper, microplasma (−) is guaranteed by the supplier (Online methods, cell culture, and transfection section) (https://www.dsmz.de/catalogues/details/culture/ACC305.html?tx_dsmzresources_pi5%5BreturnPid%5D = 192). Also, we exclusively use cells that are regularly checked for bacterial contamination.

Transfection (Supplementary Table 12): In 10 cm dishes, 1.25 × 10^5^/mL of HEK-293T cells were seeded and cultivated in medium A at 37 °C in a humidified atmosphere containing 5% CO_2_ during 43 h prior to transfection. To prepare the transfection mix, 19.2 μL of pSP27 (69.9 ng/μL, 1300 ng), 12.5 μL of pYO1 (641 ng/μL, 7800 ng), and 1.7 μL of pSEAP2-control (561 ng/μL, 900 ng) were added to 1 mL of medium B. The resulting solution was mixed with 50 μL of polyethylenimine (PEI, 20000 MW, Polysciences; stock solution 1 mg/mL in dH_2_O) and incubated for 15 min at ambient temperature. At least 30 min before addition of the prepared DNA-PEI mixture (1 mL) to the cells, the cell culture medium A was replaced by medium C. After 8 h incubation, the cell culture medium was replaced with fresh medium C. The transfected cells were split into a collagen-coated 24-well cell culture plate with a two times diluted concentration and cultivated overnight. The resulting 24-well plates were used for subsequent experiments.

### Intracellular catalysis of ArM **2**_*x*_**5**_*y*_ ⊂ Sav and FACS analysis

ArMs **2**_*x*_**5**_*y*_ ⊂ Sav (Supplementary Tables 4–9) were diluted by a factor 50 with medium B. A stock of the ruthenium complex **1** (2 mM in DMSO) was also diluted with medium B to 0.17–2.0 μM. The cell culture medium C of the aforementioned 24-well plate with the designer HEK-293T cells was replaced by media B containing either ArM **2**_*x*_**5**_*y*_ ⊂ Sav or ruthenium complex **1**. After 1 h incubation, the cells were washed three times with medium B containing heparin (0.1 mg/mL) to remove cell-surface-adsorbed ArM **2**_*x*_**5**_*y*_ ⊂ Sav as previously reported^46^. The washed cells were incubated in the medium C containing AM-AT_3_ **8** (4 μM) with 0.5% DMSO for 24 h. Expression of sec-nluc and SEAP was quantified in the supernatant as described in the following section. Then, cells were detached using 0.05% trypsin-EDTA. The cell populations were analyzed using a Becton Dickinson LSRII Fortessa Flow Cytometer (Becton Dickinson) (conditions: filter set for monitoring red fluorescence; 561-nm laser, mirror: 570 LP, Filter: 586/15 BP). Data were analyzed by FlowJo. Populations of living cells (23963 to 25336 events) were extracted from 30,000 events based on SSC and FSC.

### Activity assays of SEAP and sec-nluc

SEAP: The supernatant was transferred into a 96-well plate (100 μL/well) and incubated at 65 °C for 30 min to heat-inactivate endogenous alkaline phosphatases. After cooling, 80 μL of the treated supernatant was mixed with 100 μL of 2× SEAP buffer (20 mM homoarginine, 1 mM MgCl_2_, 21% (v/v) diethanolamine, pH 9.8) and 20 μL of 720 μM of *p*-nitrophenylphosphate. Immediately thereafter, the absorbance was measured at 405 nm at 37 °C using the EnVision 2104 Multilabel Reader. The measurement was performed over 61 repeats of 30 s/repeat and the plate was shaken for 5 s at 900 rpm at the beginning. From the time-course increase of the absorbance at 405 nm, derived from the enzymatic activity of SEAP, the activity of SEAP (in U/L) was determined. To avoid saturation effects, the linear part of the time-course plots was used for the determination of the slope. The correlation between the slope and the corresponding activity was determined using a standard.

Sec-nluc: The supernatant was transferred into a black 384-well plate (7.5 µL/well), and the same amount of nanoglo assay solution (Promega). After 5 min incubation, the luminescence was measured with Tecan Infinite M1000 Pro.

### Data availability

The authors declare that all data supporting the findings of this study are available either in the paper and in the Supplementary Information or from the authors upon reasonable request.

## Electronic supplementary material

Supplementary Information
