# Supplementary material for: A cell-penetrating artificial metalloenzyme regulates a gene switch in a designer mammalian cell
Source: Nat Commun. 2018 May 16;9:1943. doi: 10.1038/s41467-018-04440-0 (PMC5955986; doi:10.1038/s41467-018-04440-0)
Supplement: Supplementary file 1 — Supplementary Information [file 41467_2018_4440_MOESM1_ESM.pdf]

Supplementary Information

A Cell-Penetrating Artificial Metalloenzyme Regulates a Gene Switch in a Designer Mammalian Cell

Okamoto *et al.*,

Supplementary Figures

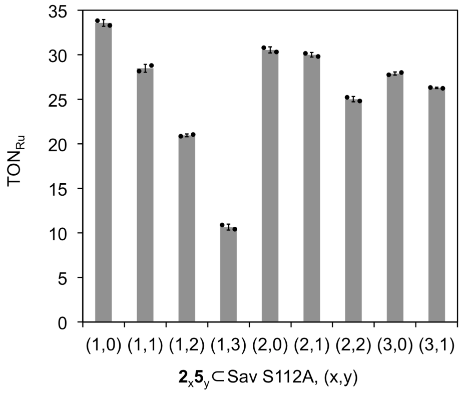

Supplementary Figure 1. Catalytic activity of ArM $\mathbf{2_x5_y}$⊂Sav for the uncaging of AT_3 6. To confirm the influence of the ratio of the ruthenium catalyst **2** vs. CPD **5** on the catalytic activity upon incorporation in homotetrameric Sav, ArMs $\mathbf{2}_x\mathbf{5}_y$⊂Sav S112A with varying x : y ratios were tested for the uncaging of AT_3 **6**. Data are the means ± standard deviation of two independent experiments. The ruthenium concentration in the reaction mixture was set to 1 μM. According to Supplementary Table 1-9, the ArMs $\mathbf{2}_x\mathbf{5}_y$⊂Sav S112A of varying x : y ratios were prepared. The reactions were performed at 37 °C in an HPLC vial using the conditions listed in Supplementary Table 11. Potassium isocyanoacetate (500 mM in water, 4 μL), Tryptophanamide hydrochloride **15** (10 mM in water, 20 μL) as an internal standard, acetonitrile (800 μL) and PBS (400 μL) were added to the reaction mixture. The resulting solution was centrifuged (10000 g) and analyzed by UPLC-MS (Supplementary Figure 19). TONs were determined using the calibration curve of T_3 **7** (Supplementary Figure 20)

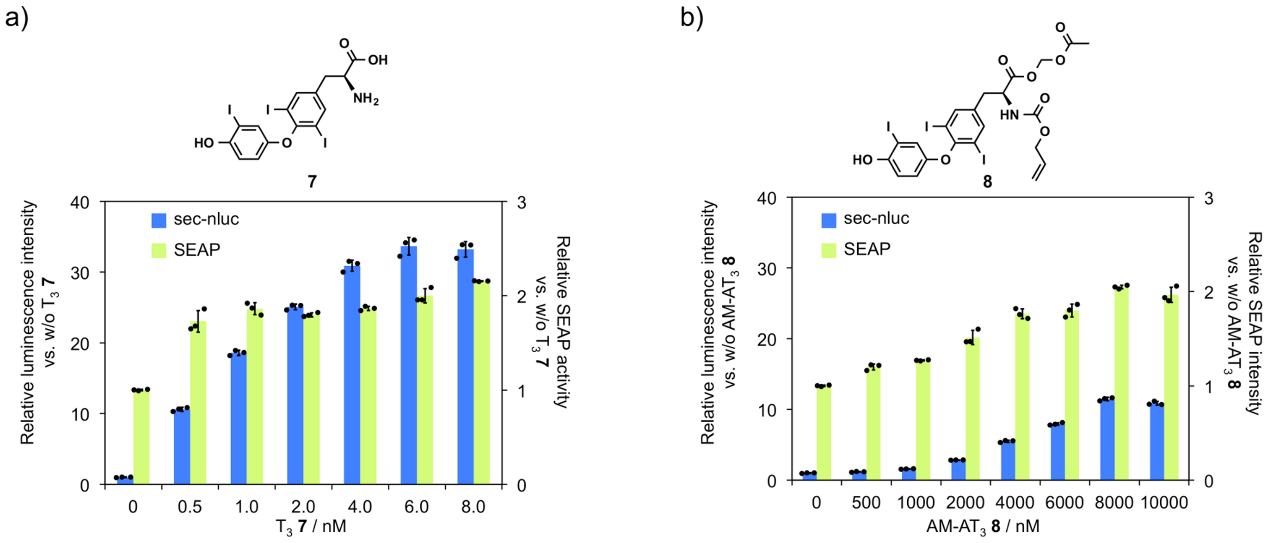

Supplementary Figure 2. Dose-dependency of T_3 7 and AM-AT_3 8 on the gene switch. Activities of sec-nluc (blue) and and SEAP (green) expressed in the HEK-293T cells transfected with pSP29, pYO1 and pSEAP2-control, 24 h after addition of (a) T_3 **7** and (b) AM-AT_3 **8**. Data are the means ± standard deviation of the three independent experiments. (See also Supplementary Method)

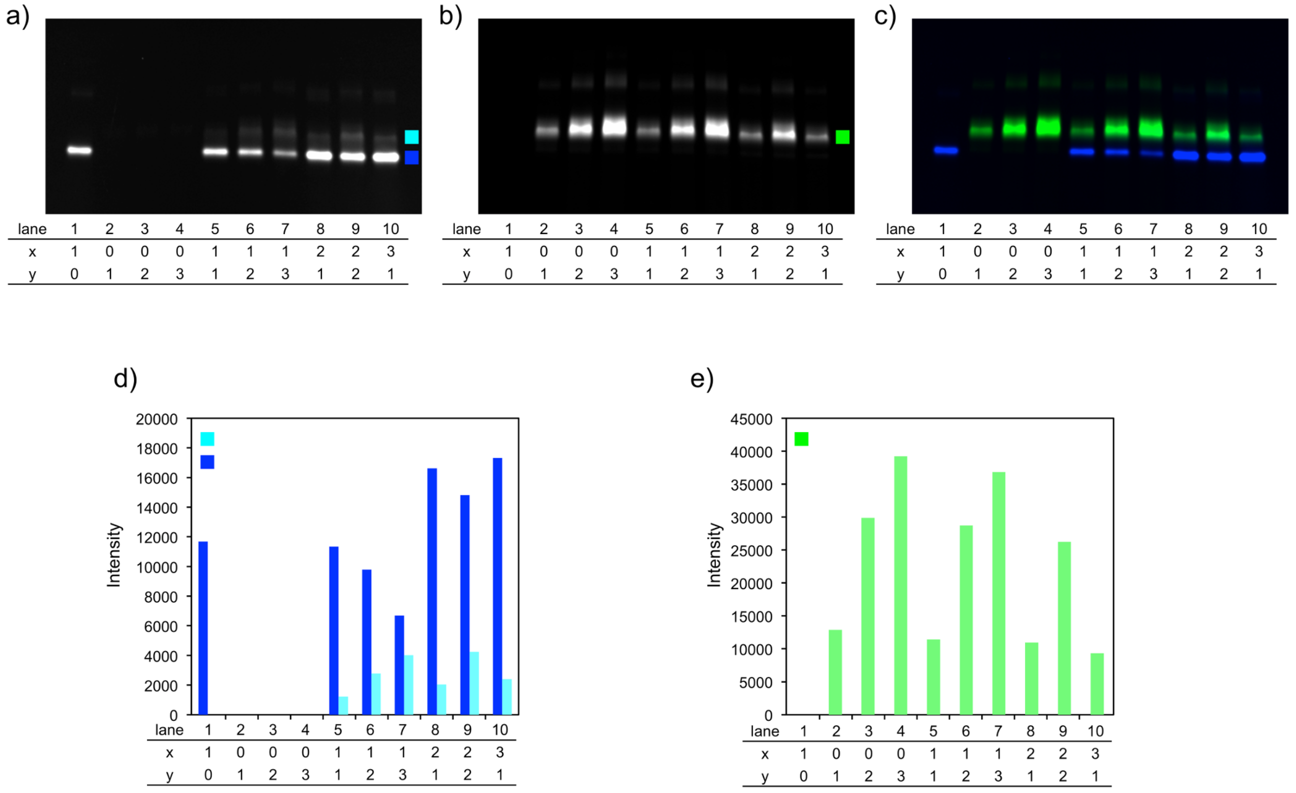

Supplementary Figure 3. Analysis of the formation of ArM $2_x5_y \subset$ Sav by SDS-PAGE. To determine whether two different biotinylated probes can be anchored within a tetrameric Sav host (0.6 μM Sav), samples containing (B4F)$_x$**5**$_y$ $\subset$ Sav (0-1.9 μM B4F (biotin-4-fluoroscein) and 0-1.9 μM CPD **5**) were analyzed by SDS-PAGE. The gel was visualized with (a) the 530/28 nm emission filter/blue Epi illumination, (b) the 650/50 nm emission filter/green Epi illumination. (c) Overlap image of (a) and (b). Intensities of (d) B4F and (e) TAMRA in CPD **5** analyzed by Image J. The upper and lower bands, which are labeled as cyan and blue squares in (a), correspond to the cyan and blue bars of (d). The band in (b) labeled as a green square corresponds to the green bars of (e). (See also Supplementary Discussions)

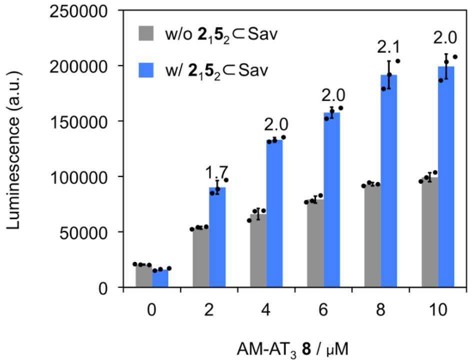

Supplementary Figure 4. Determination of the appropriate concentration of AM-AT_3 8. determine the most appropriate concentration of AM-AT_3 **8** to minimize the impact of the background luminescence caused by AM-AT_3 **8**, intracellular catalysis with $\mathbf{2}_1\mathbf{5}_2$⊂Sav (0.25 μM **2**, 0.5 μM CPD **5** and 0.25 μM Sav) was investigated. The sec-nluc activities secreted from the HEK cells transfected with pSP27, pYO1, and pSEAP2-control were compared by luminescence derived from the enzymatic activity of the sec-nluc at 24 h after treatment with (blue) and without (gray) $\mathbf{2}_1\mathbf{5}_2$⊂Sav. The concentrations of the ruthenium catalyst **2** and CPD **5** were set to 0.25 μM and 0.5 μM respectively. Starting at 4 μM AM-AT_3 **8**, the luminescence in the presence of $\mathbf{2}_1\mathbf{5}_2$⊂Sav reaches its maximum. Data are the means ± standard deviation of the three independent experiments. The numbers on the blue bars indicate the relative luminescence compared to the background luminescence observed in the absence of $\mathbf{2}_1\mathbf{5}_2$⊂Sav. (See also Supplementary Method)

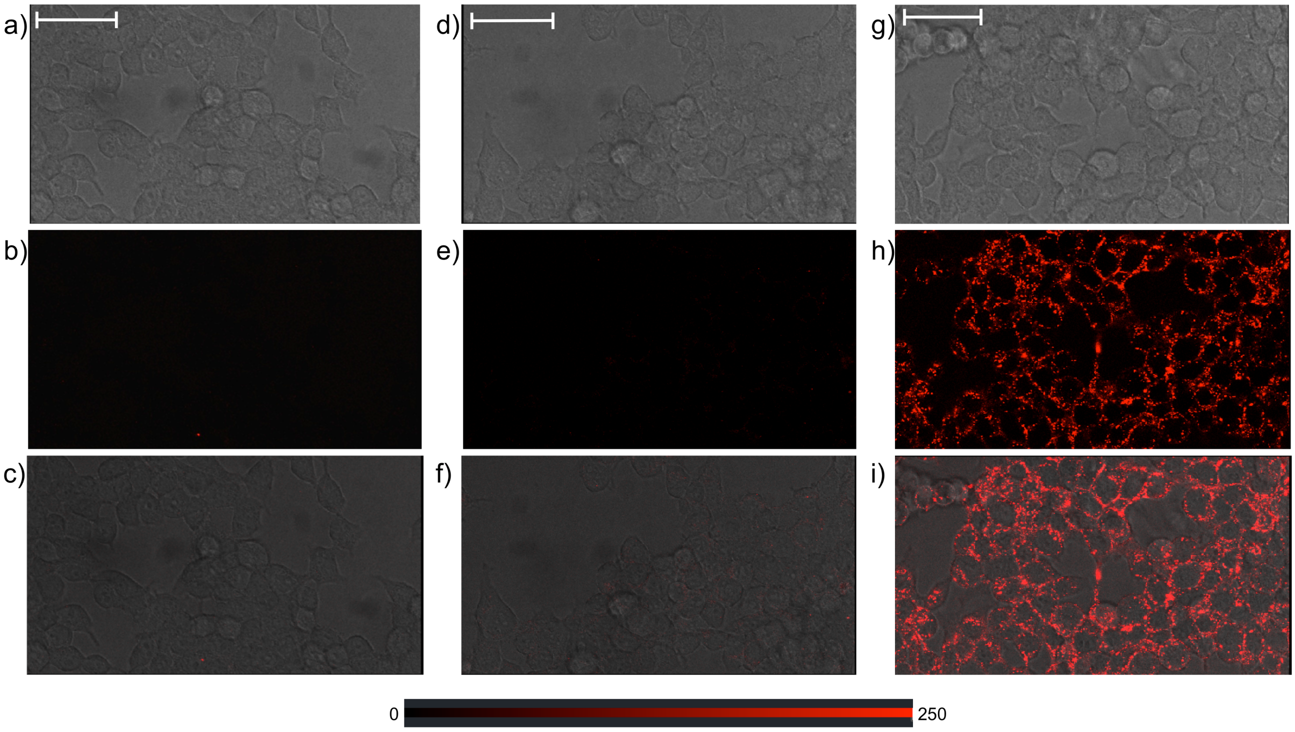

Supplementary Figure 5. Intracellular uptake of ArMs monitored by microscopy. Relying on confocal microscopy (excitation at 561 nm, emission at 571 ~ 650 nm), the HEK cells transfected with pSP27, pYO1, and pSEAP2-control were visualized one hour after treatment with the ArMs**.** Confocal microscopy images (a, d, g: differential interference contrast; b, e, h: fluorescence; c, f, i: overlay) of (a – c) designer HEK-293T cells treated with (d – f) ArM $\mathbf{2}_1$(Atto565-Biotin)$_2$⊂Sav and (g – i) ArM $\mathbf{2}_1\mathbf{5}_2$⊂Sav. The scale bars in (a), (d), and (g) indicate 50 μm. (See also Supplementary Method)

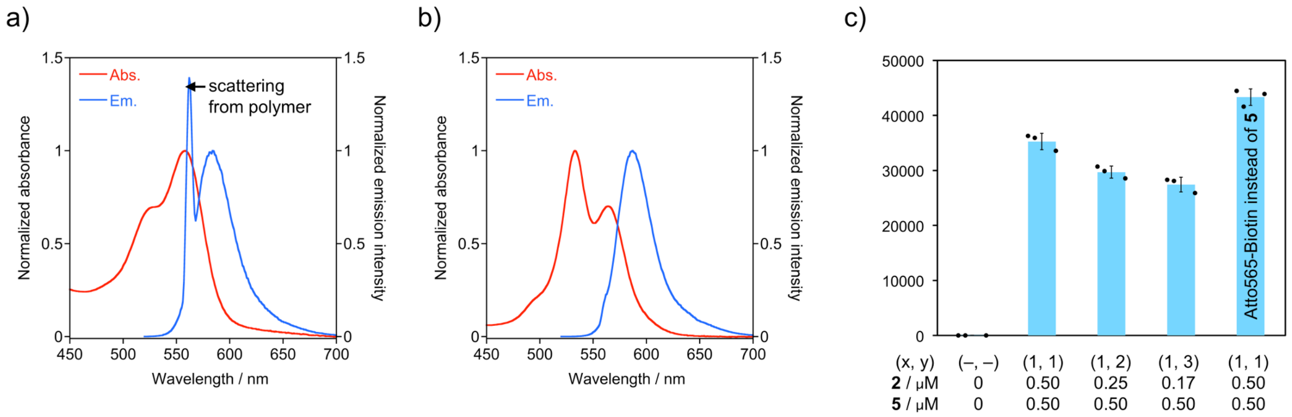

Supplementary Figure 6. Validation of the use of Atto565-biotin instead of CPD 5. In Figure 4d and in Supplementary Figure 5, Atto565-biotin was used instead of CPD **5** to highlight that CPD **5** is essential for the cell-uptake. To underscore the similarities of spectroscopic features of Atto565-biotin and CPD **5**, fluorescence of ArM $\mathbf{2}_1\mathbf{5}_1 \subset$Sav and ArM $\mathbf{2}_1$(Atto565-Biotin)$_1 \subset$Sav were measured. Normalized absorption (red) and emission (blue) spectra excited at 561 nm for (a) ArM $\mathbf{2}_1\mathbf{5}_1 \subset$Sav and (b) ArM $\mathbf{2}_1$(Atto565-Biotin)$_1 \subset$ Sav in PBS (pH 7.5). Scattering observed in the emission spectrum in (a) may be caused by CPD **3** of ArM $\mathbf{2}_1\mathbf{5}_1 \subset$Sav. The spectra were recorded by means of Fluorolog-322 from Horiba Jobin-Yvon. (c) Comparison of the fluorescent intensities (ex. at 561 nm and em. at 585 nm) of ArM $\mathbf{2}_x\mathbf{5}_y \subset$ Sav and ArM $\mathbf{2}_1$(Atto565-Biotin)$_1 \subset$ Sav (1.0 μM in PBS). Fluorescence intensities were recorded by means of Tecan Infinite M1000 Pro. Data are the means ± standard deviation of three experiments. The numbers of biotinylated catalyst moieties **2** and **5** added to the tetrameric Sav scaffold is described as (x, y). (See also Supplementary Discussions)

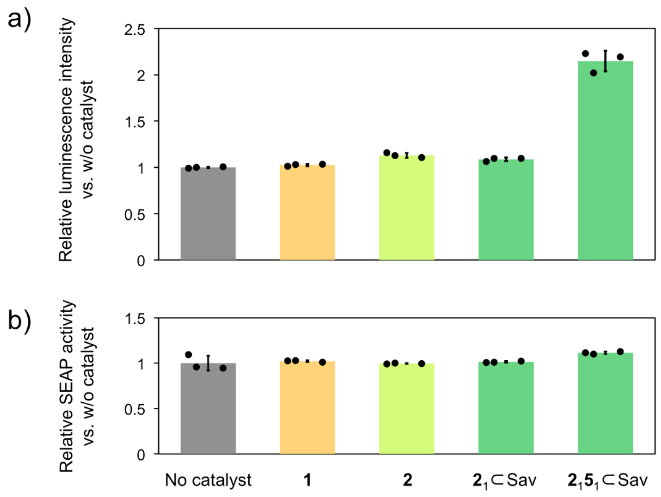

Supplementary Figure 7. Critical importance of each component contained in 2_15_1⊂ Sav. The intracellular reaction was also tested with **2**, and $\mathbf{2}_1$⊂Sav, not displayed in Figure 4e. Variation of the relative activities of expressed (a) sec-nluc and (b) SEAP secreted from the HEK cells transfected with pSP27, pYO1, and pSEAP-control2 24 h after treatment with catalysts (**1**, **2**, $\mathbf{2}_1$⊂Sav and $\mathbf{2}_1\mathbf{5}_1$⊂Sav) compared to a blank devoid of catalyst (gray) after 24 h. The ruthenium concentration was set to 0.5 μM. Data are the means ± standard deviation of the three independent experiments. (See also Supplementary Discussions)

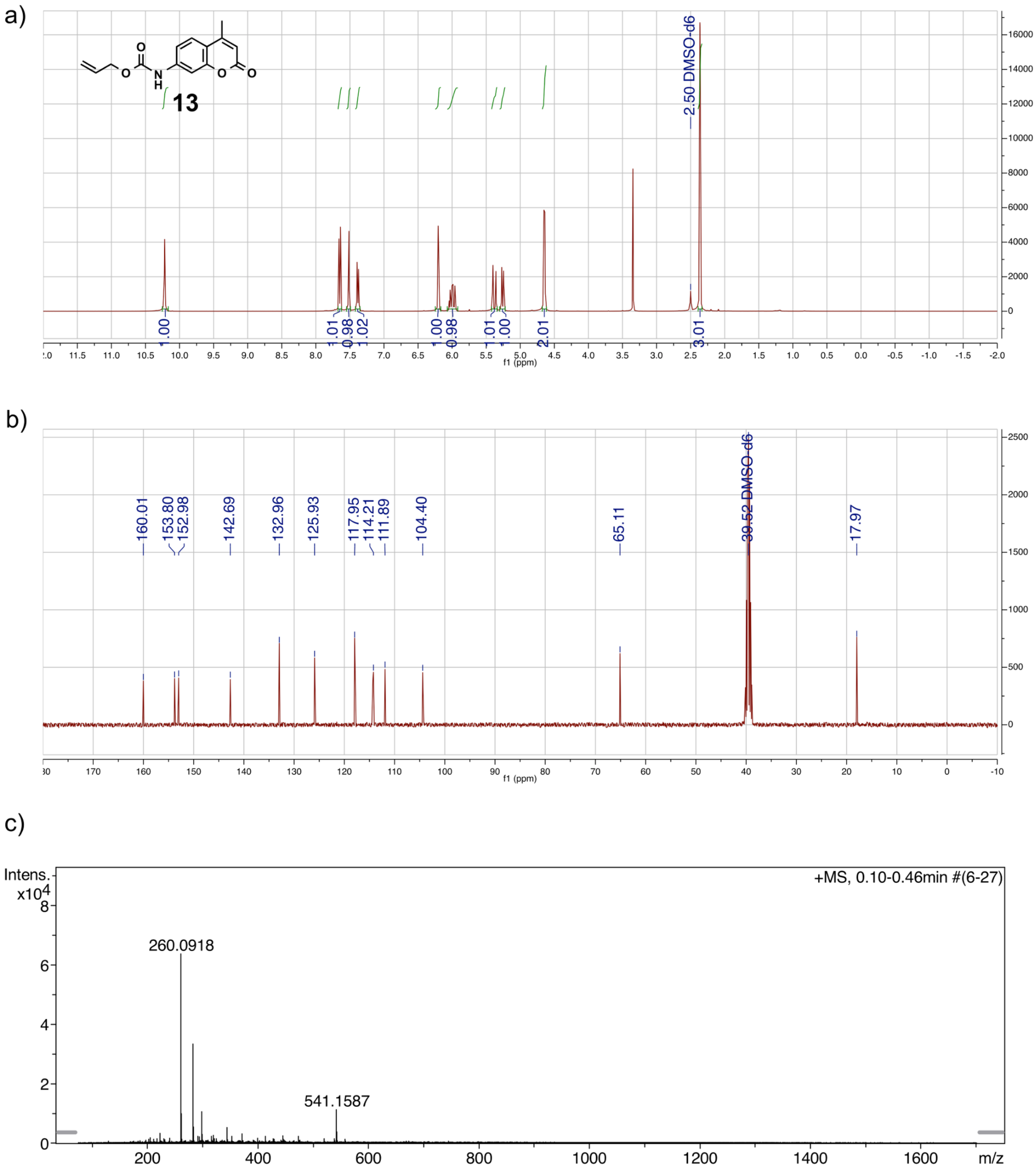

Supplementary Figure 8. Analytic data for the caged coumarin 13. (a) ^{1}H-NMR, (b) ^{13}C-NMR and (c) HRMS [ESI(+)TOF] of caged coumarin **13**. (See also Supplementary Method)

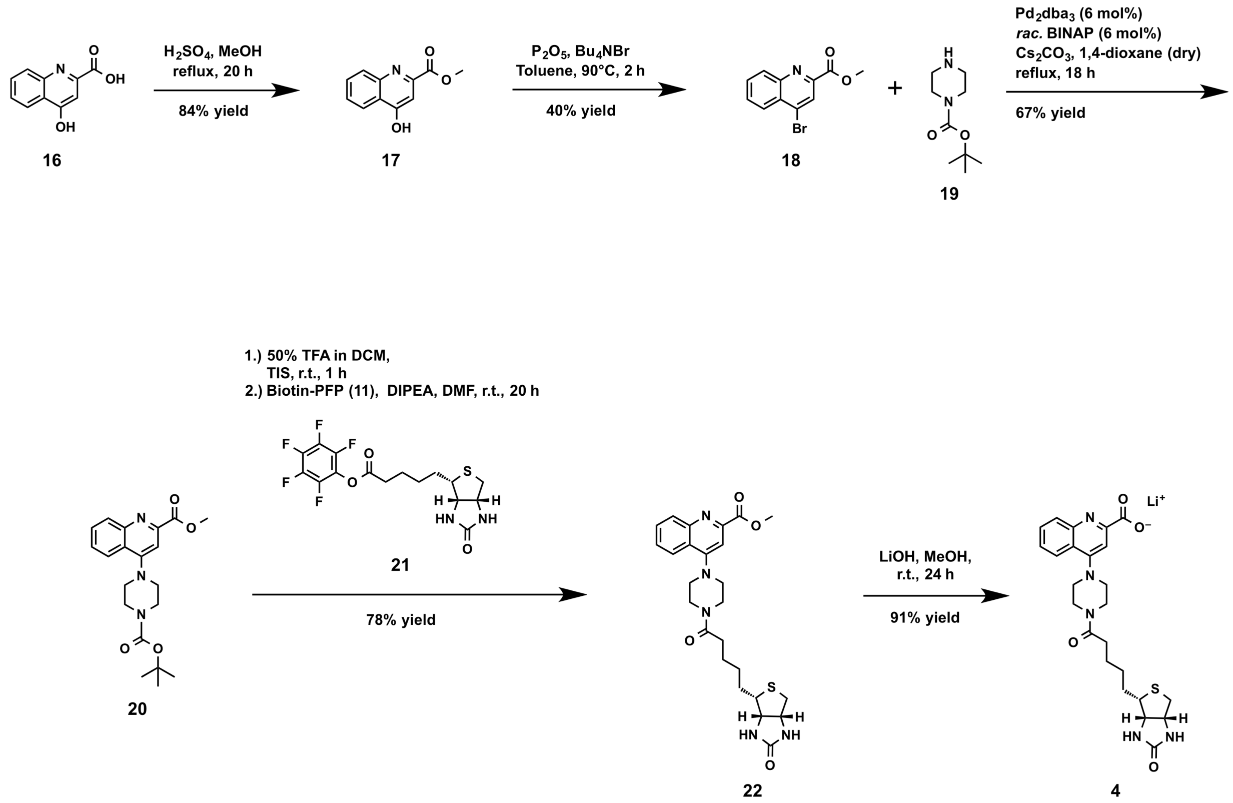

Supplementary Figure 9. Synthesis of the biotinylated ligand 4. The biotinlylated ligand **4** was synthesized following this route. Please see Supplementary Figures 10 – 15 and Supplementary Methods for the detailed synthesis and spectroscopic characterization.

a)

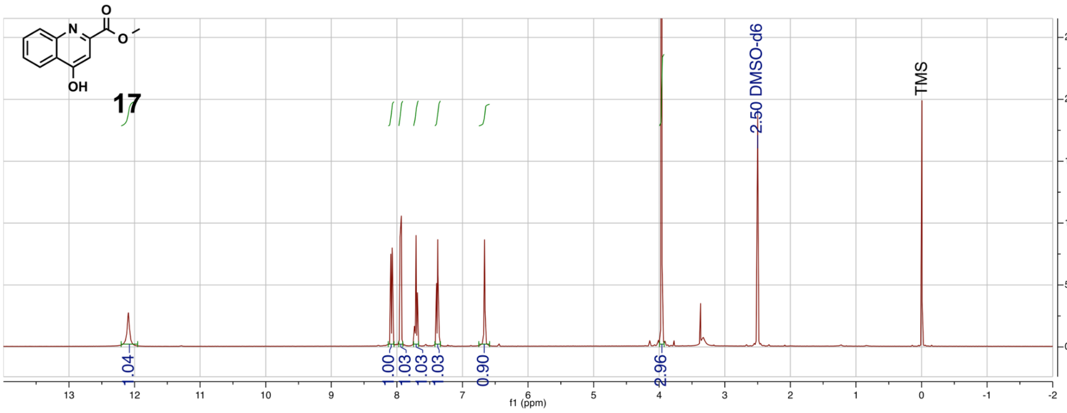

b)

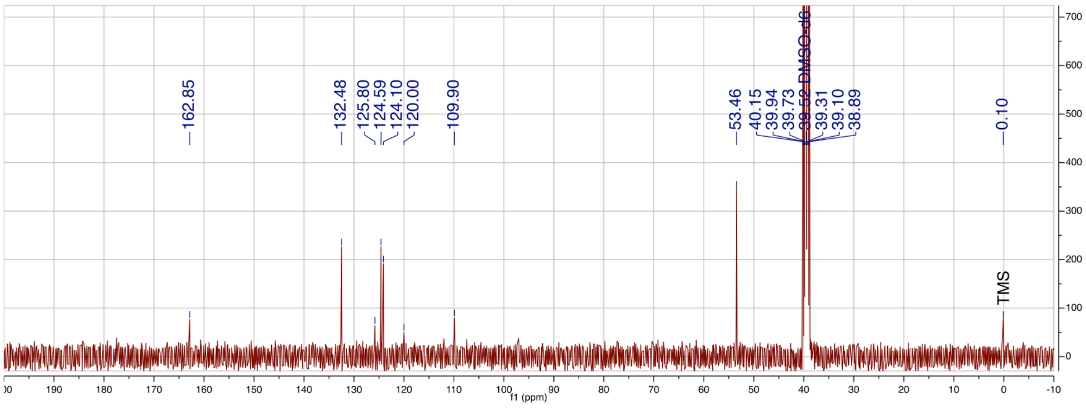

c)

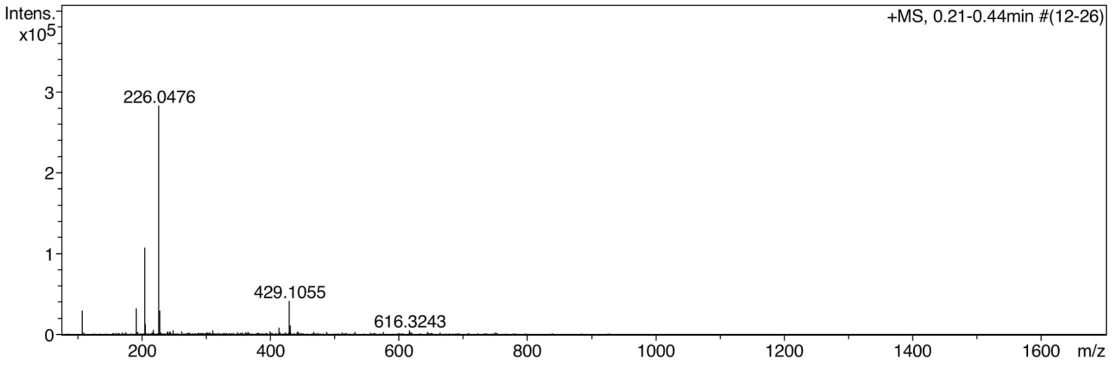

Supplementary Figure 10. (a) ^{1}H-NMR, (b) ^{13}C-NMR and (c) HRMS [ESI(+)TOF] of compound **17**. (See also Supplementary Method)

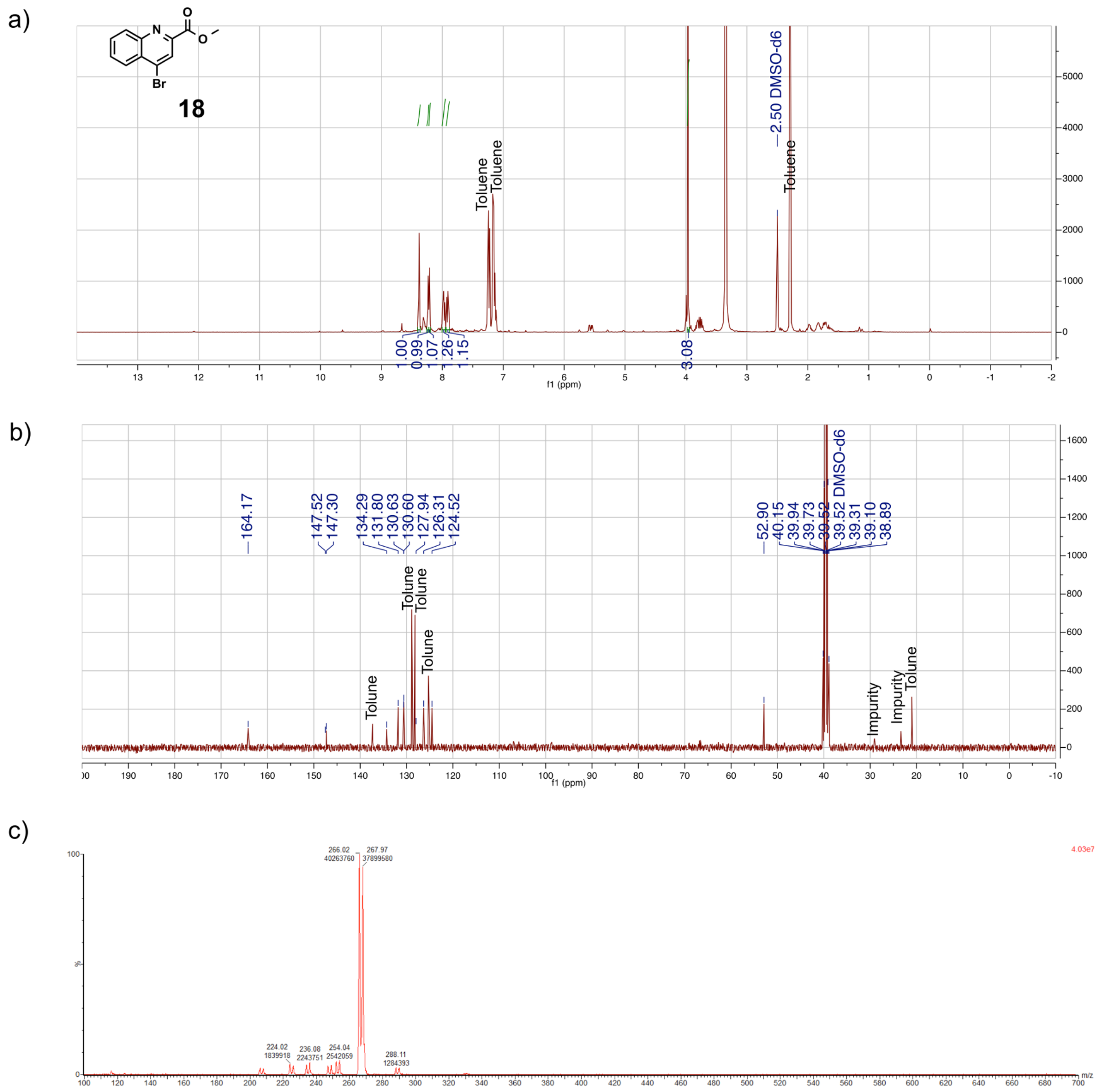

Supplementary Figure 11. (a) ^{1}H-NMR, (b) ^{13}C-NMR and (c) UPLC-MS [ESI(+)TOF] of compound **18**. (See also Supplementary Method)

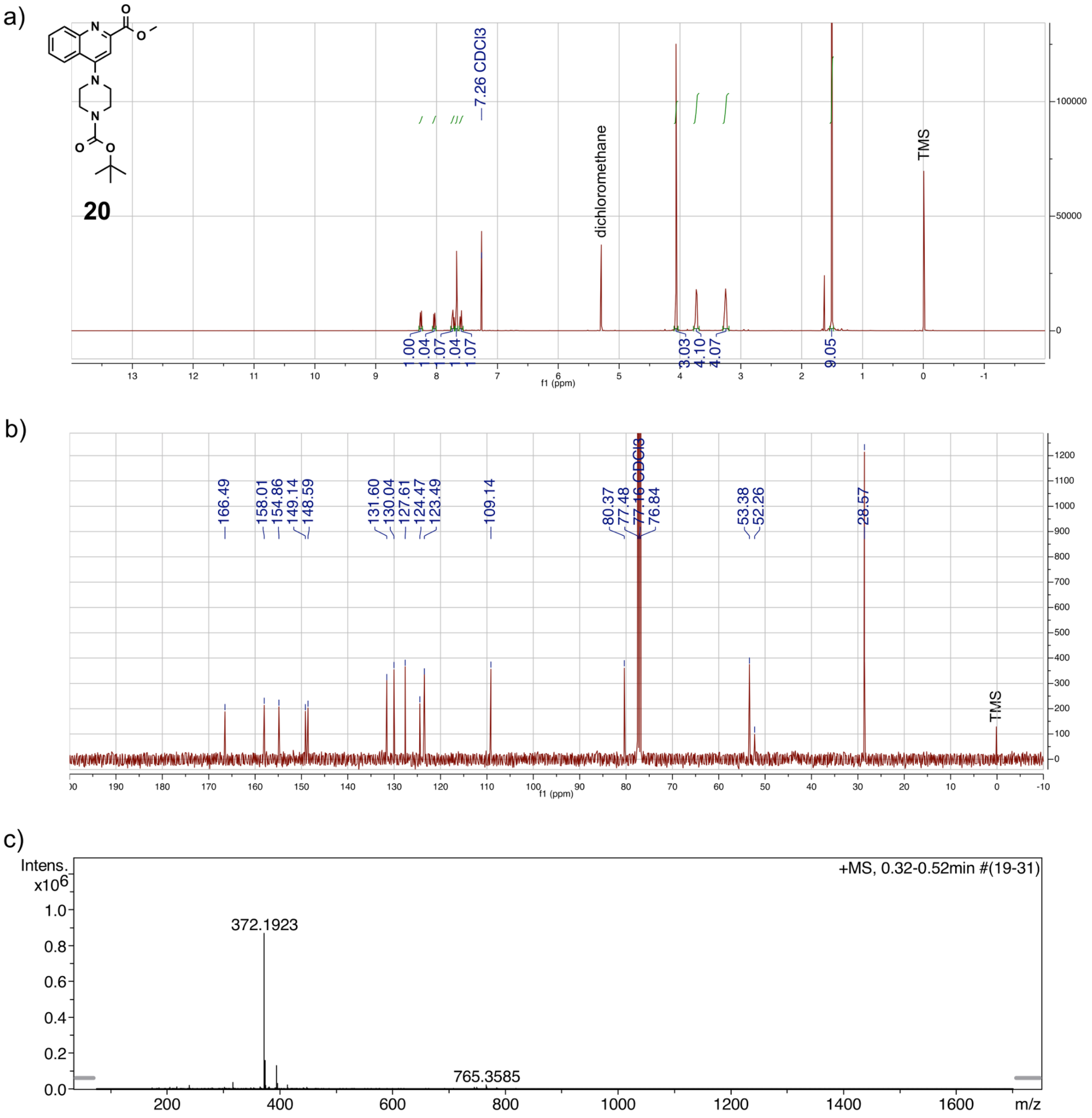

Supplementary Figure 12. (a) ^{1}H-NMR, (b) ^{13}C-NMR and (c) HRMS [ESI(+)TOF] of compound **20**. (See also Supplementary Method)

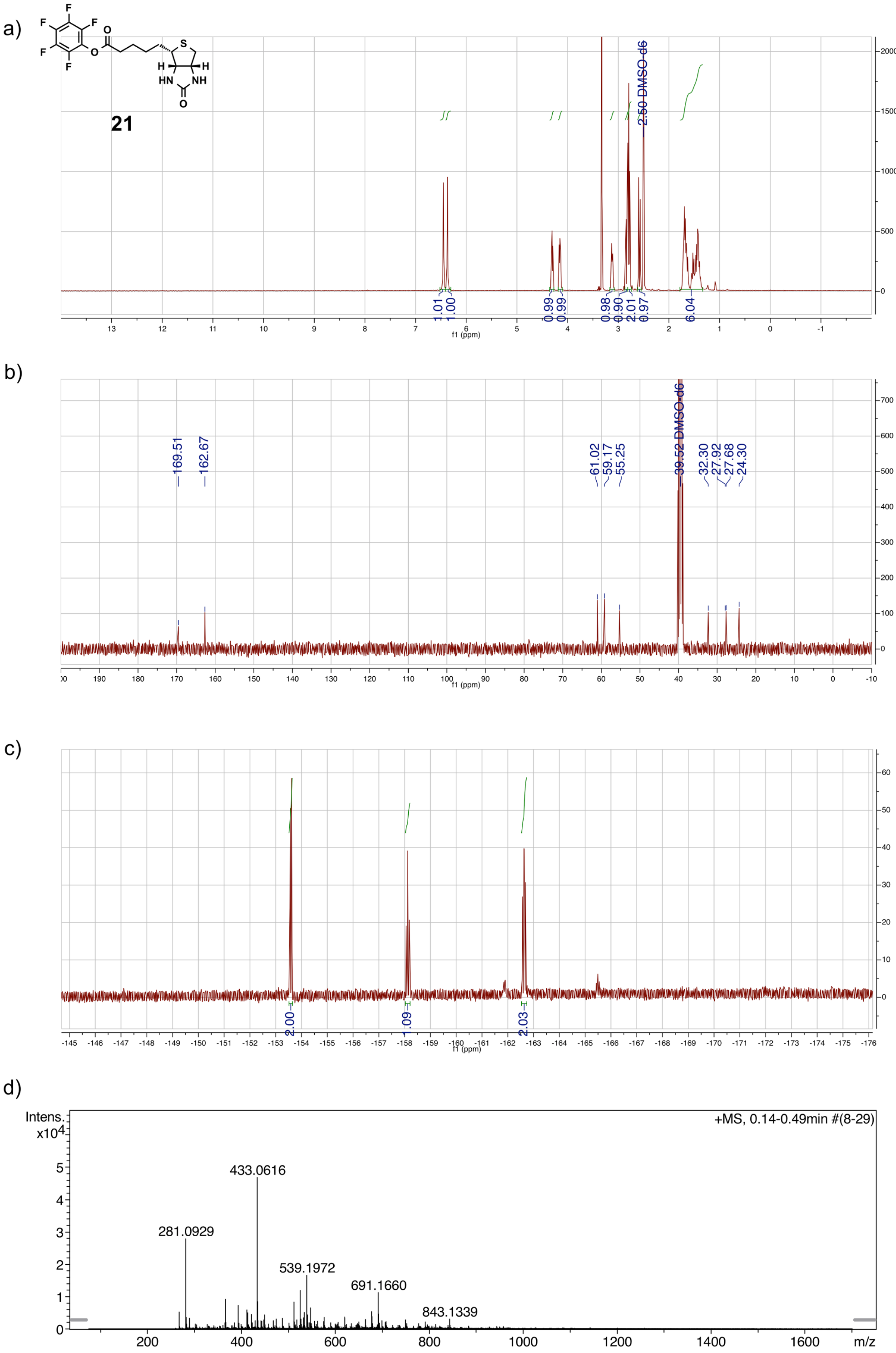

Supplementary Figure 13. (a) ^{1}H-NMR, (b) ^{13}C-NMR, (c) ^{19}F-NMR and (d) HRMS [ESI(+)TOF] of compound **21**. (See also Supplementary Method)

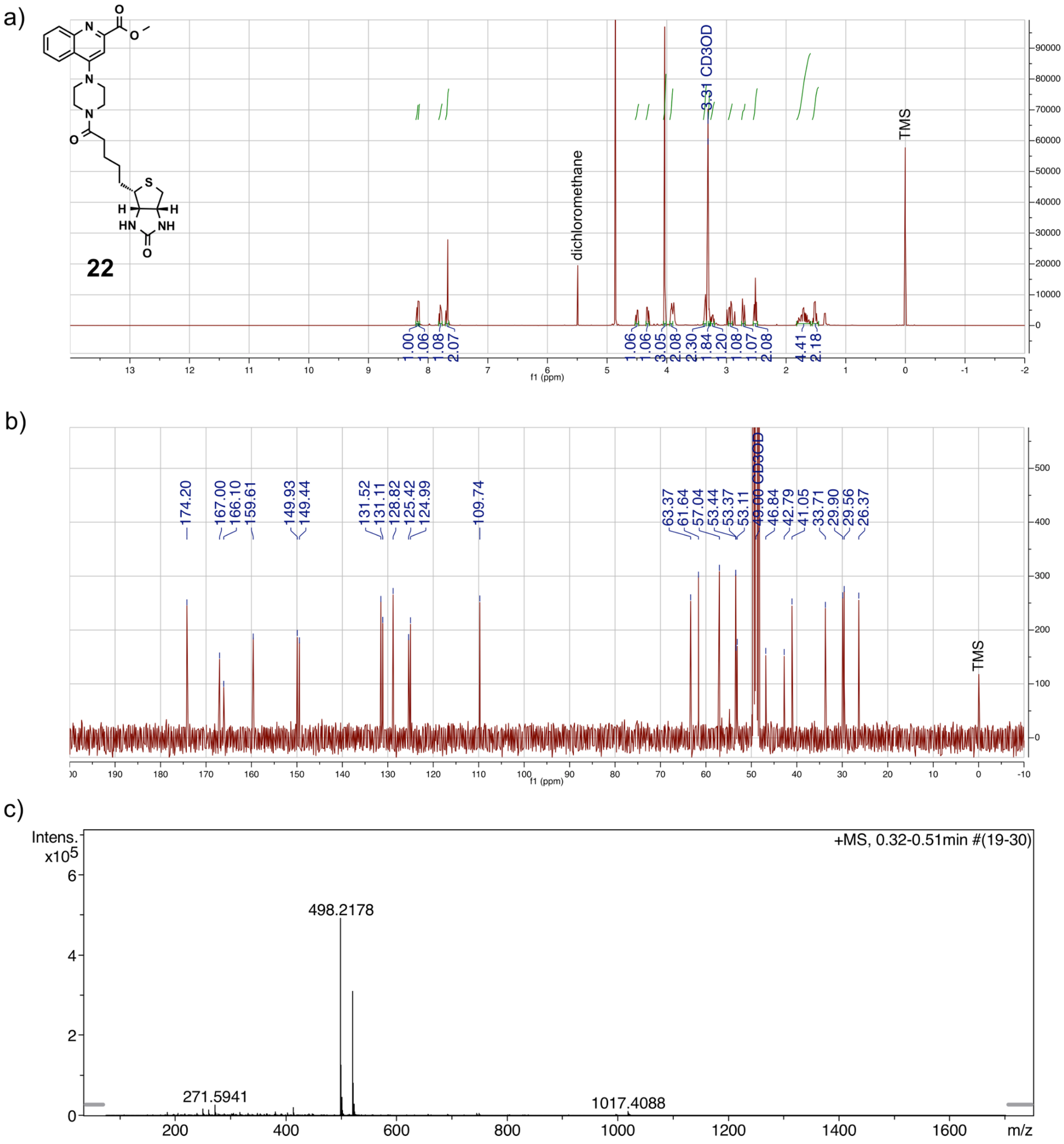

Supplementary Figure 14. (a) ^{1}H-NMR, (b) ^{13}C-NMR and (c) HRMS [ESI(+)TOF] of compound **22**. (See also Supplementary Method)

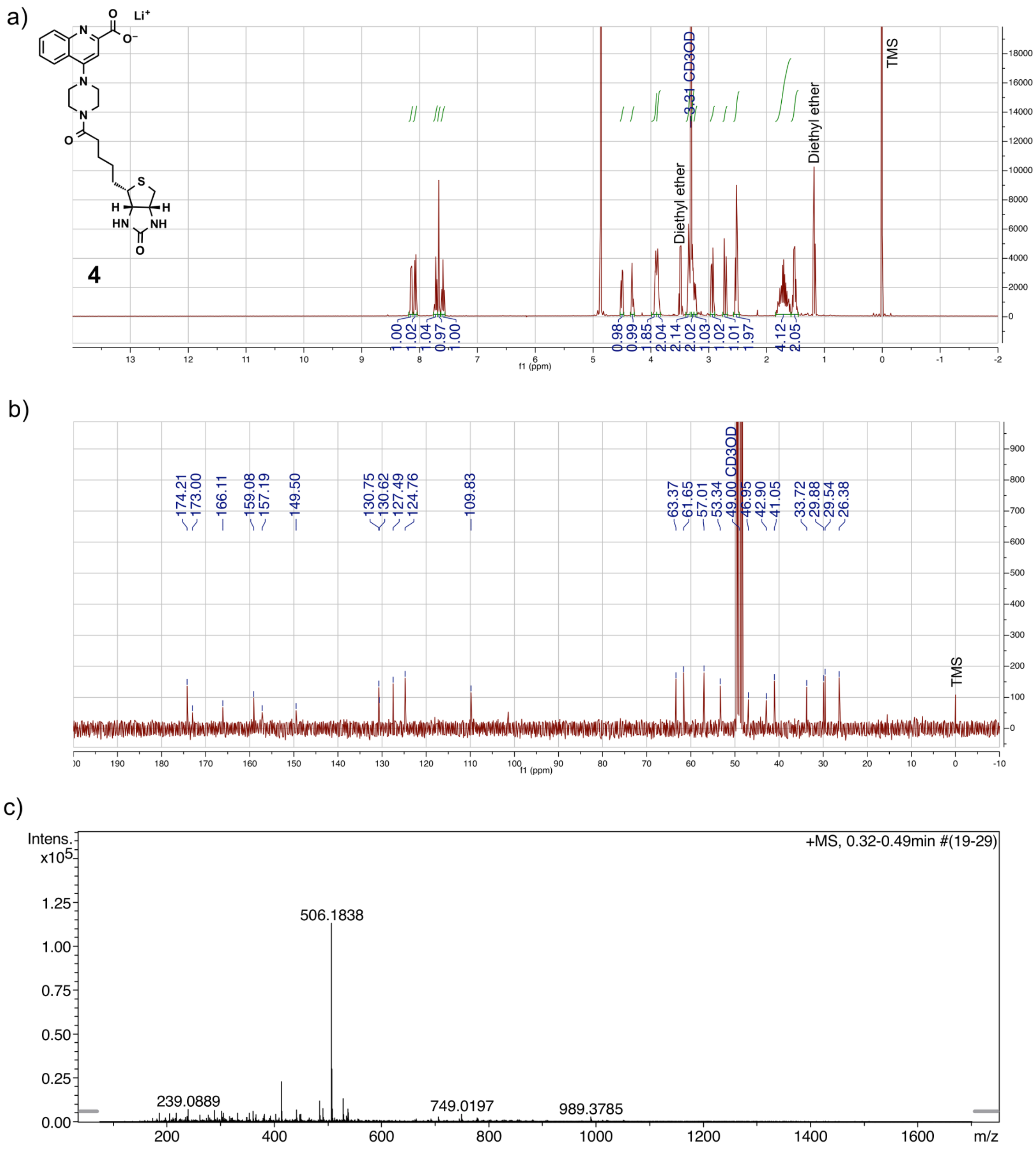

Supplementary Figure 15. (a) ^{1}H-NMR, (b) ^{13}C-NMR and (c) HRMS [ESI(+)TOF] of compound **4**. (See also Supplementary Method)

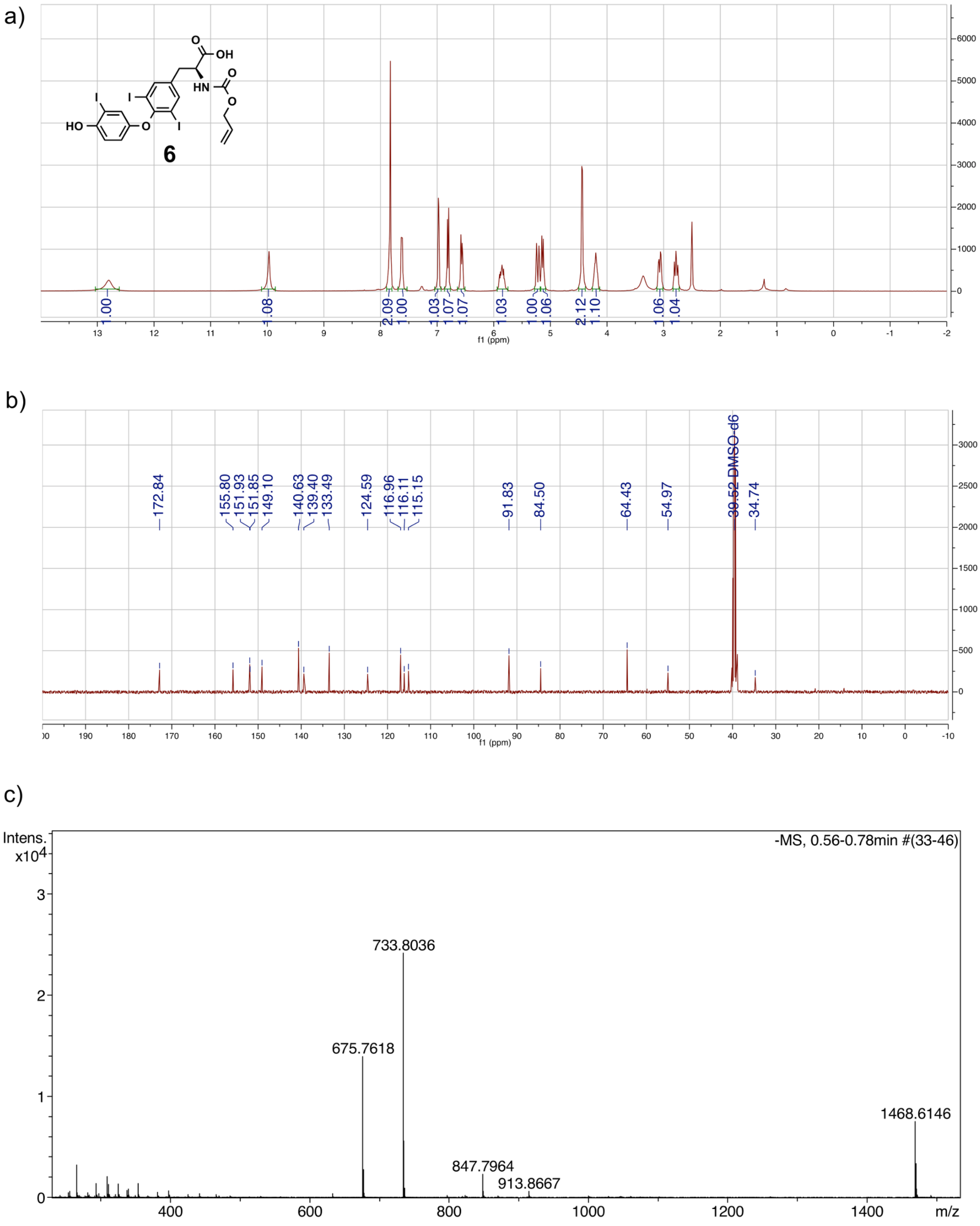

Supplementary Figure 16. Assignments of AT$_3$ 6. (a) ^{1}H-NMR, (b) ^{13}C-NMR and (c) HRMS [ESI(–)TOF] spectrum of AT$_3$ **6**. (See also Supplementary Method)

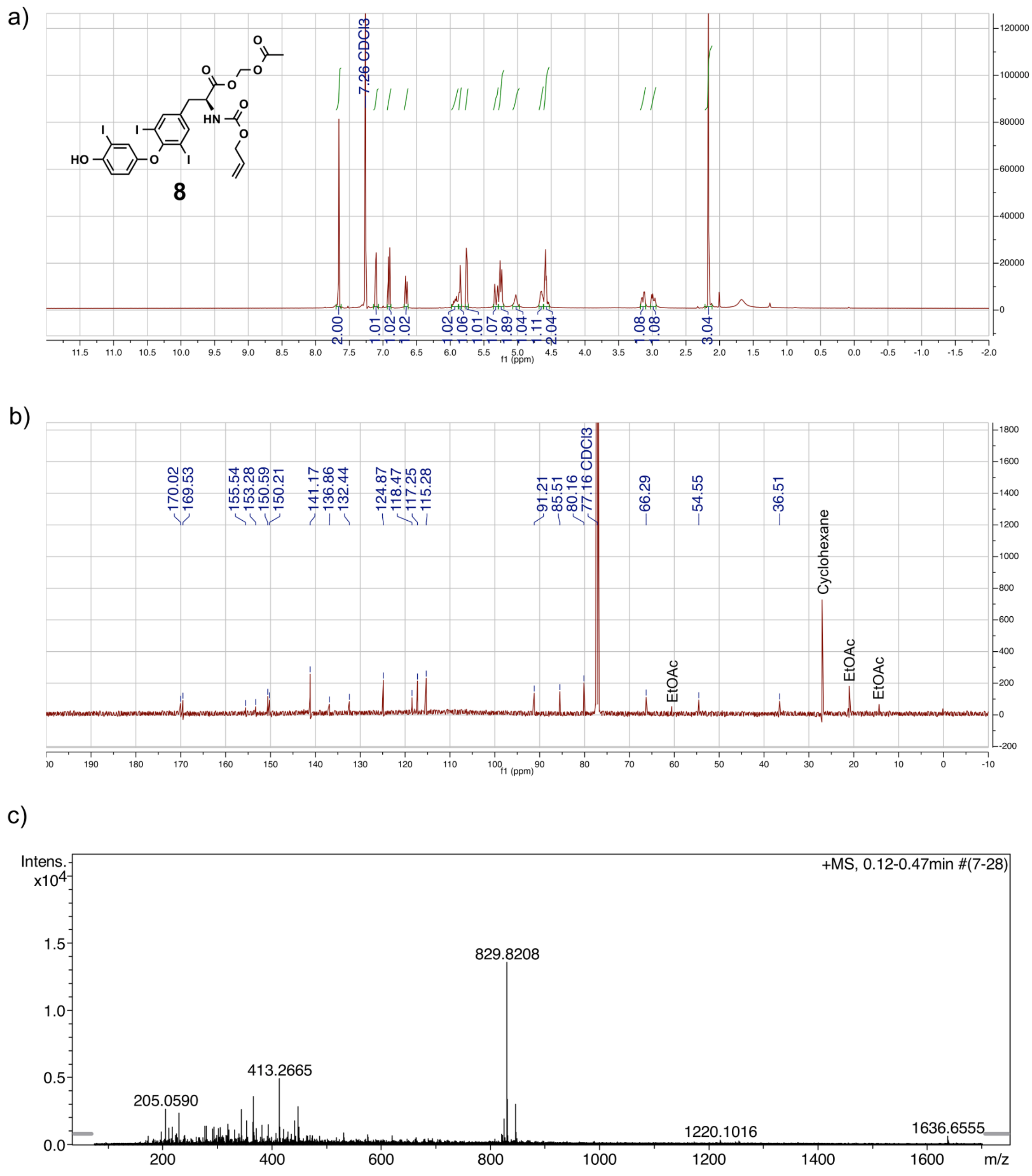

Supplementary Figure 17. Assignments of AM-AT$_3$ 8. (a) ^{1}H-NMR, (b) ^{13}C-NMR and (c) HRMS [ESI(+)TOF] spectrum of AM-AT$_3$ **8**. (See also Supplementary Method)

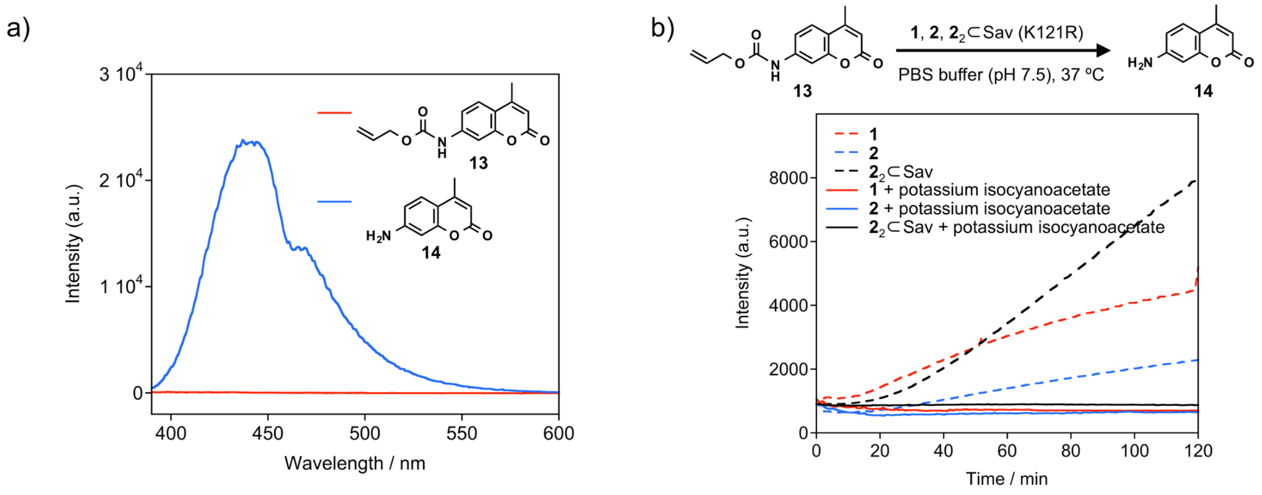

Supplementary Figure 18. Potassium isocyanoacetate as an inhibitor of Ru-catalysts. For the screening of the Sav mutants to optimize the ArM towards the uncaging of AT_3 **6** (Figure 3), an inhibitor of the ruthenium catalysts was identified to quench the reaction. To confirm that potassium isocyanoacetate acts as the inhibitor, the uncaging of substrate **13** was monitored by fluorescence of coumarin derivative **14**. (a) Fluorescence spectra of 7-amino-4methylcoumarin **14** (blue, 10 μM) and its caged precursor **13** (red, 10 μM) excitation at 375 nm in MOPS buffer (0.1 M, pH 7.0) containing 1 % DMSO. (b) Time-course evolution of the fluorescence determined at 450 nm. In the absence of potassium isocyanoacetate (dashed line), the *O*-allyl carbamate cleavage of **13** is catalyzed by the ruthenium complex **1** (red), complex **2** (blue) and ArM $\mathbf{2_2}$⊂Sav K121R (black). In the presence of potassium isocyanoacetate (solid lines), no reaction is observed, highlighting its inhibitory effect on the ruthenium catalysts. Fluorescence was recorded by means of TECAN Infinite M1000Pro. (For the experimental conditions, see the Methods section of Screening Sav for the uncaging reaction of AT_3 **6** in the main text and Supplementary Table 10.)

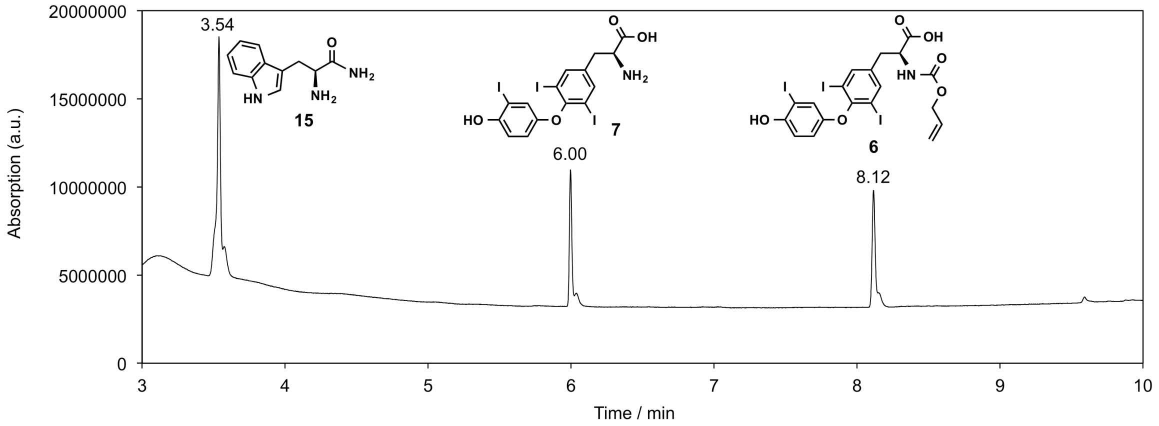

Supplementary Figure 19. **UPLC chromatogram of AT$_3$ 6 and T$_3$ 7.** A mixture of tryptophanamide **15**, AT$_3$ **6** and T$_3$ **7** were analyzed using a reversed-phase UPLC-MS. The integrated absorption from 190 – 500 nm is displayed.

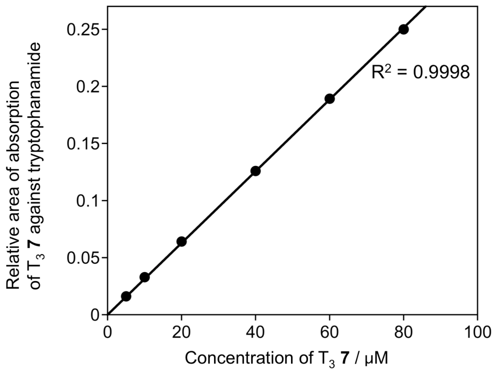

Supplementary Figure 20. Calibration curve used for the quantification of T_3 7. A calibration curve of the T_3 **7** was determined relying on the relative area of the absorption of T_3 **7** against tryptophanamide **15** added as an internal standard. (See also Supplementary Figure 19)

Supplementary Tables

Supplementary Table 1. Stock solution of ArMs $\mathbf{2}_3 \subset$Sav

Stock solution	Initial conc. (µM)	Volume (µL)	Final conc. (µM)
Stock **A**	800 (FBBS)	83.3	133.3
Stock **C**	5000	10.0	100.0
Water		406.7	
		total 500 µL	

Supplementary Table 2. Stock solution of ArMs $\mathbf{2}_2 \subset$Sav

Stock solution	Initial conc. (µM)	Volume (µL)	Final conc. (µM)
Stock **A**	800 (FBBS)	125.0	200.0
Stock **C**	5000	10.0	100.0
Water		365.0	
		total 500 µL	

Supplementary Table 3. Stock solution of ArMs $\mathbf{2}_1 \subset$Sav

Stock solution	Initial conc. (µM)	Volume (µL)	Final conc. (µM)
Stock **A**	800 (FBBS)	250.0	400.0
Stock **C**	5000	10.0	100.0
Water		240.0	
		total 500 µL	

Supplementary Table 4. Stock solution of ArMs $\mathbf{2}_3\mathbf{5}_1 \subset$Sav

Stock solution	Content	Initial conc. (µM)	Volume (µL)	Final conc. (µM)
ArMs $\mathbf{2}_3 \subset$Sav	Sav	133.3 (FBBS)	15.0	100.0
	Ru **2**	100.0		75.0
CPD **5**	CPD **5**	110.0	4.5	25.0
Water			0.5	
			total 20 µL	

Supplementary Table 5. Stock solution of ArMs $\mathbf{2}_2\mathbf{5}_2\subset$Sav

Stock solution	Content	Initial conc. (μM)	Volume (μL)	Final conc. (μM)
ArMs $\mathbf{2}_2\subset$Sav	Sav	200.0 (FBBS)	5.0	50.0
	Ru **2**	100.0		25.0
CPD **5**	CPD **5**	110.0	4.5	25.0
Water			10.5	
			total 20 μL	

Supplementary Table 6. Stock solution of ArMs $\mathbf{2}_2\mathbf{5}_1\subset$Sav

Stock solution	Content	Initial conc. (μM)	Volume (μL)	Final conc. (μM)
ArMs $\mathbf{2}_2\subset$Sav	Sav	200.0 (FBBS)	10.0	100.0
	Ru **2**	100.0		50.0
CPD **5**	CPD **5**	110.0	4.5	25.0
Water			5.5	
			total 20 μL	

Supplementary Table 7. Stock solution of ArMs $\mathbf{2}_1\mathbf{5}_1\subset$Sav

Stock solution	Content	Initial conc. (μM)	Volume (μL)	Final conc. (μM)
ArMs $\mathbf{2}_1\subset$Sav	Sav	400.0 (FBBS)	5.0	100.0
	Ru **2**	100.0		25.0
CPD **5**	CPD **5**	110.0	4.5	25.0
Water			10.5	
			total 20 μL	

Supplementary Table 8. Stock solution of ArMs $\mathbf{2}_1\mathbf{5}_3\subset$Sav

Stock solution	Content	Initial conc. (μM)	Volume (μL)	Final conc. (μM)
ArMs $\mathbf{2}_1\subset$Sav	Sav	400.0 (FBBS)	1.7	33.3
	Ru **2**	100.0		8.3
CPD **5**	CPD **5**	110.0	4.5	25.0
Water			13.8	
			total 20 μL	

Supplementary Table 9. Stock solution of ArMs $\mathbf{2}_1\mathbf{5}_2\subset$Sav

Stock solution	Content	Initial conc. (μM)	Volume (μL)	Final conc. (μM)
ArMs $\mathbf{2}_1\subset$Sav	Sav	400.0 (FBBS)	2.5	50.0
	Ru **2**	100.0		12.5
CPD **5**	CPD **5**	110.0	4.5	25.0
Water			13.0	
			total 20 μL	

Supplementary Table 10. Conditions for the deprotection of **13** in a 96-well plate

Stock solution	Content	Initial conc. (μM)	Volume (μL)	Final conc. (μM)
ArMs $\mathbf{2}_2\subset$Sav[a]	Sav	200.0 (FBBS)	10.0	10.0
	Ru **2**	100.0		5.0
Caged coumarin **13** (in DMSO)		10000.0	2.0	100.0
Potassium isocyanoacetate		500000	2.0[b]	5000
PBS (pH 7.5)			186	
			total 200 μL	

[a] Stock solution **B** for **1** and stock solution **C** for **2** were diluted to 100 μM with PBS. The diluted samples were used instead of ArM. [b] PBS was used for the experiments without potassium isocyanoacetate.

Supplementary Table 11. Conditions for the *O*-allyl carbamate cleavage of AT_3 **6**

Stock solution	Content	Initial conc. (μM)	Volume (μL)	Final conc. (μM)
ArMs $\mathbf{2}_2\subset$Sav[a]	Sav	200.0 (FBBS)	4.0	2.0
	Ru **2**	100.0		1.0
AT_3 **6** (in DMSO)		2000.0	20.0	100.0
PBS (pH 7.5)			376	
			total 400 μL	

[a] Stock solution **B** for **1** and stock solution C for **2** were diluted to 100 μM with PBS. The diluted samples were used instead of ArM.

Supplementary Table 12. Plasmids used in this study.

Plasmid	Description and Cloning strategy	Reference / Source
**pSEAP2-control**	Constitutive SEAP expression vector (P_{SV40}-SEAP-pA_{SV40})	Clontech
**pSP27**	Constitutive TSR expression vector (P_{hCMV}-TSR-pA_{bGH})	Saxena *et al.* [1]
**pYO1**	P_{UAS5} driven secreted nanoluc (sec-nluc) expression vector (P_{UAS5}-sec-Nluc-pA_{bGH}). Sec-nluc was PCR- amplified from pRK0[2] by using the following primers: oRK187 (TAATaagcttgccaccATGGAGACAGACACACTCCTGCTATGGGTACTGCTGCTCTGGGTTCCAGGTTCCACTGGTGACgtcttcacactcgaagatttcgttggggac) and oRK188 (ATGCtctagattacgccagaatgcgttcgcac). The amplified fragment was digested with *Hind*III/*Xba*I and was cloned into a plasmid bearing the same multicloning site as pcDNA4 V5-His B, yielding pRK154. The sequence of pRK154 was confirmed by a reverse primer for bGH-pA (TAGAAGGCACAGTCGAGG). Then, a fragment coding for sec-nluc was cut out from pRK154 with *Nhe*I/*Age*I, and was cloned into pSP29 (PUAS5-SEAP-pAbGH) digested with *Xba*I/*Age*I/*Sac*II, replacing SEAP with sec-nluc. (Note that *Xba*I and *Nhe*I are compatible ends. *Sac*II was added to readily distinguish the non-necessary fragment). The successful integration of the sec-nluc into the pYO1 plasmid was confirmed as follows: i) digestion with the restriction enzymes (*Eco*RI/*Age*I) and agarose gel-electrophoresis reveals a band with the right size (695 bp) and ii) transfection of the pYO1 plasmid in HEK-293T cells in the presence of the T_3 hormone **7** leads to luminescence thus highlighting the presence of the sec-nluc gene in the pYO1 plasmid.	This work

Supplementary Discussions

Analysis of the formation of ArM $\mathbf{2_x3_y}$⊂Sav by SDS-PAGE.** (for Supplementary Figure 3)

Since biotin binding to the streptavidin is non-cooperative[3], thus affording a Poisson distribution of $\mathbf{2}_x\mathbf{5}_y$⊂Sav species, we performed SDS-PAGE to analyze the formation of Sav conjugates loaded with two different biotinylated probes. For visualization purposes by fluorescence, the commercially available biotin-4-fluoroscein (B4F) was used instead of the biotinylated complex **2**. Using a similar procedure summarized in Supplementary Table 4-9, various $(B4F)_x\mathbf{5}_y$⊂Sav constructs using a fixed Sav concentration (0.6 μM) were prepared. Despite the chaotropic SDS-PAGE conditions, Sav maintains its tetrameric nature and its biotin-binding affinity. B4F and the TAMRA moiety in CPD **5** were visualized by fluorescence upon excitation at their respective wavelength. (Supplementary Figure 3a for B4F and 3b for CPD **5**). As expected, $\mathbf{5}_y$⊂Sav is shifted toward higher molecular masses compared to Sav, Supplementary Figure 3. In Supplementary Figure 3a, two bands are observed for lanes 5-6, highlighting binding of B4F to Sav. The weak upper bands in Supplementary Figure 3a (cyan in Supplementary Figure 3a and 3d) coincide with the Sav bands bearing CPD **5** in Supplementary Figure 3b. This confirms that streptavidin binds simultaneously to B4F and CPD **5**. The weak fluorescence of the upper band in Supplementary Figure 3a is tentatively rationalized by a FRET between the TAMRA moiety in CPD **5** and B4F[4]. From Supplementary Figure 3d, the intensities of upper bands in Supplementary Figure 5a increase in the order of lane 5 ($(B4F)_1\mathbf{5}_1$⊂Sav) < 8 ($(B4F)_2\mathbf{5}_1$⊂Sav) < 10 ($(B4F)_3\mathbf{5}_1$⊂Sav). This trend confirms the results observed for the intracellular uncaging reaction of AM-AT_3 **8** with $\mathbf{2}_x\mathbf{5}_y$⊂Sav (Figure 4e).

Validation of the usage of Atto565-biotin instead of CPD 5 (for Supplementary Figure 6). Atto565-Biotin from Sigma-Aldrich was used as a control to analyze the effect of CPD **3** on the cellular uptake. The fluorescence of ArM $\mathbf{2}_1\mathbf{5}_1 \subset$Sav and ArM $\mathbf{2}_1$(Atto565-Biotin)$_1 \subset$Sav were compared (Supplementary Figure 6). *In vitro*, the fluorescence intensity of ArM $\mathbf{2}_1$(Atto565-Biotin)$_1 \subset$ Sav is roughly comparable to that of ArM $\mathbf{2}_1\mathbf{5}_1 \subset$ Sav in PBS (Supplementary Figure 6c). From the FACS results presented in Figure 3e and Supplementary Figure 6c, we conclude that the ArM $\mathbf{2}_1$(Atto565-Biotin)$_1 \subset$Sav (i.e. lacking CPD **5**) does not permeate into the cells.

Critical importance of each component contained in $\mathbf{2}_1\mathbf{5}_1 \subset$Sav. (for Supplementary Figure 7)

Different types of catalysts were investigated for the uncaging AM-AT$_3$ **8** (4 μM) in the designer HEK-293T cells transfected with pSP27, pYO1, and pSEAP2-control. The conditions are described in the Methods section of main text. Constant expression levels of SEAP among all of the uncaging experiments highlights that there is no critical cytotoxicity for the intracellular reaction. Except for $\mathbf{2}_1\mathbf{5}_1 \subset$Sav, all other tested catalysts did not lead to the upregulation of the T$_3$-responsive gene switch.

Supplementary Methods

Materials

Sav variants,[5,6] cell-penetrating poly(disulfide)s **5**[7], and the ruthenium catalyst **1**[8] were produced, purified and characterized as previously reported. The synthesis of the biotinylated ligand **4** and the substrates **6**, **8**, and **13** is presented below. Other reagents, substrates and materials were purchased from Sigma-Aldrich, TCI, Acros, Fluorochem, Invitrogen and Promega.

UV−Vis and fluorescence measurements were conducted on a Varian 50 Scan UV-vis spectrophotometer, Tecan Infinite M1000 Pro, Envision 2104 Multilabel Reader or Fluorolog-322 from Horiba Jobin-Yvon. NMR experiments were performed on a Bruker Avance III NMR spectrometer operating at 400 or 500 MHz proton frequency. Chemical shifts are referenced to residual $CHCl_3$ or $CDCl_3$ for ^1H spectra (7.26 ppm) and ^{13}C spectra (77.16 ppm) or $(D_2HC)SO(CD_3)$ (2.50 ppm) and $(D_3C)SO(CD_3)$ (39.51 ppm), respectively. High-resolution MS analyses were carried out on a Bruker maXis 4G. Cell counting was performed using a Casy® TTC Cell Counter. FACS measurements were conducted by BD LSRFortessa. Fluorescence imaging was performed with a Leica SP5. SDS-PAGE was visualized using a Bio-rad ChemiDoc™ MP imaging system and analyzed by ImageJ. UPLC-MS measurements were performed with an Acquity UPLC H Class Bio from Waters equipped with a PDA and a SQ detector 2 with the following column: ACQUITY UPLC, HSS T3 1.8 µm, 2.1 x 100 mm. Solvents were water and acetonitrile, respectively, each containing 0.1 % formic acid, referred to as (A) and (B) respectively. The flow rate was set to 0.60 ml/min and the temperature to 40 °C. Method 1: 0 min – 100% A; 1.21 min – 100% A; 10 min – 10% A. A Water Prep LC 4000 System equipped with a Waters 2487: Dual λ Absorbance Detector as UV-Vis detector was used for preparative separations with the following column: XSelect CSH Prep C18 OBD, 19 x 150 mm, 5 µm.

Synthesis of compound 17 (for Supplementary Figure 10**)**

Kynurenic acid **16** (5.0 g, 26.4 mmol, 1.0 eq.) was dispersed in dry methanol (50 ml, 39.6 g, 1240 mmol, 47 eq.). Concentrated sulfuric acid (3.0 ml, 5.5 g, 56.3 mmol, 2.1 eq.) was added, whereupon the mixture became clear. The solution was heated to reflux for 20 h under an N_2 atmosphere. The solution was evaporated to dryness, yielding yellow oil. Water (100 ml) and saturated $NaHCO_3$ (100 ml) were added, whereupon an off-white solid precipitated. The solid was filtered, washed with diethylether (50 ml) and dried to yield a white solid (**17**, 4.53 g, 22.3 mmol, 84%).

^{1}H NMR (400 MHz, DMSO-d$_6$) δ 12.09 (s, 1H), 8.08 (dd, *J* = 8.1, 1.5 Hz, 1H), 7.94 (d, *J* = 8.4 Hz, 1H), 7.71 (ddd, *J* = 8.5, 7.0, 1.6 Hz, 1H), 7.38 (ddd, *J* = 8.1, 7.0, 1.1 Hz, 1H), 6.66 (s, 1H), 3.96 (s, 3H).

^{13}C NMR (101 MHz, DMSO-d$_6$) δ 162.85, 140.45 (extracted from HMBC spectrum), 132.48, 125.80, 124.59, 124.10, 120.00, 109.90, 53.46.

HRMS [ESI(+)TOF]: calculated for $C_{11}H_{10}NO_3$ $[M+H]^+$ 204.0655; found: 204.0655

Synthesis of compound 18 (for Supplementary Figure 11)

A mixture of compound **17** (2.30 g, 11.3 mmol, 1.0 eq.), P_2O_5 (3.55 g, 24.9 mmol, 2.2 eq.) and Bu_4NBr (4.02 g, 12.4 mmol, 1.1 eq.) in toluene (80 ml) was heated at 90 °C for 1 h with vigorous stirring. After cooling to room temperature, the resulting upper toluene layer was carefully separated. The organic phase was washed with saturated $NaHCO_3$ (150 ml), brine (150 ml) and water (150 ml), dried over Na_2SO_4 and concentrated under reduced pressure to yield a yellow solid (**18**, 1.10 g, 4.12 mmol, 36%). The crude product was used for the next step without further purification.

^{1}H NMR (400 MHz, DMSO-*d*$_6$) δ 8.38 (s, 1H), 8.23 (s (broad), 1H), 8.21 (s (broad), 1H), 8.01 – 7.94 (m, 1H), 7.94 – 7.88 (m, 1H), 3.97 (s, 3H).

^{13}C NMR (101 MHz, DMSO-*d*$_6$) δ 164.17, 147.52, 147.30, 134.29, 131.80, 130.63, 130.60, 127.94, 126.31, 124.52, 52.90.

UPLC-MS [ESI(+)TOF]: calculated for $C_{12}H_8{}^{79}BrNO_2$ $[M+H]^+$ 265.98, found: 266.02; calculated for $C_{12}H_8{}^{81}BrNO_2$ $[M+H]^+$ 267.98, found: 267.97

Synthesis of compound 20 (for Supplementary Figure 12)

Under an N_2 atmosphere compound **18** (1.00 g, 3.76 mmol, 1.0 eq.), *tert*-butyl piperazine-1-carboxylate **19** (707 mg, 3.76 mmol, 1.0 eq.), $Pd_2(dba)_3$ (196 mg, 0.23 mmol, 0.06 eq.), (*rac*)-BINAP (135 mg, 0.23 mmol, 0.06 eq.) and $CsCO_3$ (2.74 g, 8.41 mmol, 2.2 eq.) were mixed with dry 1,4-dioxane (30 ml) and heated to reflux for 15 h. The red mixture was filtered and the clear solution evaporated to dryness. The resulting red oil was taken up in ethyl acetate and purified by silica gel column (flash chromatography, ethyl acetate/cyclohexane 1:2 → 1:1). The fractions were concentrated and dried under reduced pressure to yield a yellow-orange solid (**20**, 409 mg, 1.10 mmol, 29%).

^{1}H NMR (400 MHz, $CDCl_3$) δ 8.26 (ddd, *J* = 8.5, 1.3, 0.6 Hz, 1H), 8.04 (ddd, *J* = 8.4, 1.5, 0.6 Hz, 1H), 7.73 (ddd, *J* = 8.5, 6.8, 1.4 Hz, 1H), 7.67 (s, 1H), 7.59 (ddd, *J* = 8.2, 6.8, 1.3 Hz, 1H), 4.06 (s, 3H), 3.78 – 3.69 (m, 4H), 3.30 – 3.19 (m, 4H), 1.50 (s, 9H).

^{13}C NMR (101 MHz, $CDCl_3$) δ 166.49, 158.01, 154.86, 149.14, 148.59, 131.60, 130.04, 127.61, 124.47, 123.49, 109.14, 80.37, 53.38, 52.26, 43.63 (extracted from HMQC spectrum), 28.57.

HRMS [ESI(+)TOF]: calculated for $C_{20}H_{26}N_3O_4$ $[M+H]^+$ 372.1918, found: 372.1923

Synthesis of compound 21 (for Supplementary Figure 13)

To a dispersion of D-biotin (1.00 g, 4.08 mmol, 1.0 eq.) in DMF (25 ml) triethylamine (1.00 ml, 0.73 g, 7.17 mmol, 1.8 eq.) was added at 0 °C. Pentafluorophenyl trifluoroacetate (1.00 ml, 1.63 g, 5.81 mmol, 1.4 eq) was slowly added, which led to the formation of a pink solution. The reaction mixture was allowed to warm up to room temperature and was further stirred for 2 h, whereupon a white precipitate formed. Diethylether (80 ml) was added, the white solid was filtered, washed with diethylether (80 ml) and dried at reduced pressure to yield the product as a white solid (**21**, 998 mg, 2.43 mmol, 60%).

^{1}H NMR (400 MHz, DMSO-d_6) δ 6.45 (s, 1H), 6.37 (s, 1H), 4.36 – 4.27 (m, 1H), 4.19 – 4.11 (m, 1H), 3.17 – 3.08 (m, 1H), 2.87 – 2.81 (m, 1H), 2.79 (t, *J* = 7.6 Hz, 2H), 2.58 (d, *J* = 12.4 Hz, 1H), 1.78 – 1.34 (m, 6H).

^{13}C NMR (101 MHz, DMSO-d_6) δ 169.51, 162.67, 61.02, 59.17, 55.25, 39.78 (extracted from HMQC spectrum), 32.30, 27.92, 27.68, 24.30.

^{19}F NMR (376 MHz, DMSO-d_6) δ -153.59 (d, *J* = 19.1 Hz, 2F), -158.12 (t, *J* = 23.1 Hz, 1F), -162.63 (dd, *J* = 23.3, 19.1 Hz, 2F).

HRMS [ESI(+)TOF]: calculated for $C_{16}H_{15}N_2O_3SF_5Na$ $[M+Na]^+$ 433.0616, found: 433.0616

Synthesis of compound 22 (for Supplementary Figure 14)

Compound **20** (0.40 g, 1.09 mmol, 1.0 eq.) and triisopropylsilane (0.44 ml, 0.35 g, 2.18 mmol, 2.0 eq.) were dissolved in DCM (2 ml) and treated with concentrated TFA (2 ml). The red solution was stirred for 1 h at room temperature and then evaporated to dryness. The orange oil was dissolved in DCM (2 ml). Addition of diethyl ether (20 ml) led to the formation of a yellow precipitate which was filtered, washed with diethyl ether (2 x 10 ml) and dried under reduced pressure to obtain an off-white solid (0.52 g). This solid was dissolved in DMF (5 ml), followed by the addition of *N*,*N*-diisopropylethylamine (0.95 ml, 0.70 g, 5.44 mmol, 5.0 eq.) and D-biotin pentafluorophenyl ester (**21**, 0.44 g, 1.09 mmol, 1.0 eq.). The reaction mixture was stirred for 24 h at room temperature (until no more D-biotin pentafluorophenyl ester was detectable on TLC (DCM/MeOH 10:1)) and evaporated to dryness to yield a brown oil. Addition of diethyl ether (20 ml) led to the precipitation of an off-white solid, which was filtered and washed with diethyl ether (4 x 50 ml). The solid was then dissolved in DCM (20 ml), washed with saturated $NaHCO_3$ (20 ml) and water (20 ml). The organic fraction was dried over Na_2SO_4. The solvent was evaporated under reduced pressure to afford a pale yellow solid (**22**, 224 mg, 0.85 mmol, 78%).

^{1}H NMR (400 MHz, Methanol-*d*$_4$) δ 8.18 (dd, *J* = 5.6, 0.8 Hz, 1H), 8.16 (dd, *J* = 5.4, 0.6 Hz, 1H), 7.79 (ddd, *J* = 8.5, 6.9, 1.4 Hz, 1H), 7.68 (ddd, *J* = 8.2, 6.8, 1.2 Hz, 1H), 7.67 (s, 1H), 4.50 (ddd, *J* = 7.9, 5.0, 1.0 Hz, 1H), 4.32 (dd, *J* = 7.9, 4.4 Hz, 1H), 4.03 (s, 3H), 3.92 (t, *J* = 4.8 Hz, 2H), 3.88 (t, *J* = 5.0 Hz, 2H), 3.39 – 3.33 (m, 2H), 3.30 – 3.27 (m, 2H), 3.23 (ddd, *J* = 8.8, 5.9, 4.4 Hz, 1H), 2.93 (dd, *J* = 12.8, 5.0 Hz, 1H), 2.71 (d, *J* = 12.6 Hz, 1H), 2.51 (t, *J* = 7.4 Hz, 2H), 1.83 – 1.59 (m, 4H), 1.56 – 1.45 (m, 2H).

^{13}C NMR (101 MHz, Methanol-*d*$_4$) δ 174.24, 167.04, 166.14, 159.64, 149.97, 149.48, 131.56, 131.15, 128.86, 125.46, 125.03, 109.78, 63.41, 61.68, 57.08, 53.48, 53.41, 53.15, 46.88, 42.83, 41.09, 33.75, 29.94, 29.60, 26.41.

HRMS [ESI(+)TOF]: calculated for $C_{25}H_{32}N_5O_4S$ $[M+H]^+$ 498.2170, found: 498.2178

Synthesis of compound 4 (for Supplementary Figure 15)

Compound **22** (100 mg, 0.20 mmol, 1.0 eq.) was dissolved in MeOH (2 ml) and treated with $LiOH{\cdot}H_2O$ (16 mg, 0.40 mmol, 2.0 eq.). The reaction mixture was stirred for 22 h at room temperature (until no more starting material was detected on TLC (DCM/MeOH 10:1)). The mixture was filtrered to remove the excess of insoluble LiOH. Addition of diethyl ether (5 ml) led to the formation of an off-white precipitate, which was washed with diethyl ether (3 x 5 ml) and dried at reduced pressure to obtain an off-white solid (**4**, 80.9 mg, 0.17 mmol, 83%).

^{1}H NMR (400 MHz, Methanol-d_4) δ 8.14 (dd, J = 8.4, 0.8 Hz, 1H), 8.07 (dd, J = 8.6, 0.9 Hz, 1H), 7.72 (ddd, J = 8.4, 6.8, 1.4 Hz, 1H), 7.66 (s, 1H), 7.59 (ddd, J = 8.3, 6.8, 1.2 Hz, 1H), 4.50 (ddd, J = 7.9, 5.0, 0.9 Hz, 1H), 4.32 (dd, J = 7.9, 4.4 Hz, 1H), 3.91 (t, J = 5.4 Hz, 2H), 3.88 (t, J = 4.7 Hz, 2H), 3.39 – 3.32 (m, 2H), 3.30 – 3.23 (m, 2H), 3.27 – 3.18 (m, 1H), 2.93 (dd, J = 12.7, 5.0 Hz, 1H), 2.71 (d, J = 12.7 Hz, 1H), 2.52 (t, J = 7.4 Hz, 2H), 1.84 – 1.58 (m, 4H), 1.57 – 1.45 (m, 2H).

^{13}C NMR (101 MHz, Methanol-d_4) δ 174.21, 173.00, 166.11, 159.08, 157.19, 149.50, 130.75, 130.62, 127.49, 124.76, 124.75, 109.83, 63.37, 61.65, 57.01, 53.34, 53.34, 46.95, 42.90, 41.05, 33.72, 29.88, 29.54, 26.38.

HRMS [ESI(+)TOF]: calculated for $C_{24}H_{30}N_5O_4S$ $[M+H_2]^+$: 484.2013, found: 484.2014

Synthesis of AT_3 6 (for Supplementary Figure 16)

3,3',5-Triiodo-L-thyronine **7** (600 mg, 0.921 mmol) was dissolved in a mixture of water (15 ml) and acetone (12 ml). To this solution, K_2CO_3 (255 mg, 1.84 mmol) and $NaHCO_3$ (77 mg, 0.921 mmol) were added. After cooling on an ice bath, allyl chloroformate (120 mg, 0.995 mmol, 1.08 eq.) was added under an N_2 atmosphere and the mixture was stirred for 3 h on ice. The reaction mixture was acidified to pH 2 by addition of HCl_{aq} (1 M) and extracted with EtOAc. The evaporated mixture was purified using silicagel column chromatography (CH_2Cl_2 : MeOH : CH_3COOH = 95 : 4 : 1) to yield 202 mg (30%) of the title compound **6** as a white solid.

^{1}H NMR (400.1 MHz, DMSO-d_6) δ 12.8 (bs, 1H), 9.97 (s, 1H), 7.83 (s, 2H), 7.62 (d, 1H, *J* = 8.6 Hz), 6.97 (d, 1H, *J* = 3.0 Hz), 6.81 (d, 1H, *J* = 8.8 Hz), 6.57 (dd, 1H, *J* = 8.8, 3.0 Hz), 5.86 (ddt, 1H, *J* = 16.0, 10.4, 5.2 Hz), 5.22 (d, 1H, *J* = 17.4 Hz), 5.14 (d, 1H, *J* = 10.5 Hz), 4.44 (d, 1H, *J* = 5.2 Hz), 4.20 (ddd, 1H, *J* = 13.4, 9.3, 4.1 Hz), 3.07 (dd, 1H, *J* = 13.9, 4.4 Hz), 2.78 (d, 1H, *J* = 13.8, 10.6 Hz)

^{13}C NMR (100.6 MHz, DMSO-d_6) δ 172.8, 155.8, 151.93, 151.85, 149.1, 140.6, 139.4, 133.5, 124.6, 117.0, 116.1, 115.2, 91.83, 84.5, 64.4, 54.97, 34.74

HRMS [ESI(–)TOF]: calculated for $C_{19}H_{15}I_3NO_6$ $[M–H]^-$ 733.8039; found 733.8036

Synthesis of AM-AT$_3$ 8 (for Supplementary Figure 17)

AT$_3$ **6** (240 mg, 0.327 mmol) and *N,N*-diisopropylethylamine (169 mg, 1.31 mmol, 4.0 eq.) were dissolved in dry acetonitrile (10 ml). To the resulting solution, bromomethylacetate (150 mg, 0.979 mmol, 3.0 eq.) dissolved in dry acetonitrile (10 ml) was added dropwise under an N_2 atmosphere. The mixture was stirred for 18 h at ambient temperature and the volatiles were evaporated under reduced pressure. The mixture was purified using C18-reverse phase HPLC with eluent A (H_2O containing 0.1 % TFA) and eluent B (acetonitrile containing 10% H_2O and 0.1 % TFA). The flow rate was set to 20 ml/min. Method 1: 0 min – 40% B; 3.4 min – 40% B; 6.4 min – 65%B; 30 min – 100%B. The collected fractions were lyophilized to yield 40 mg (14%) of the title compound **8** as a white solid.

^{1}H NMR (400.1 MHz, $CDCl_3$) δ 7.65 (s, 2H), 7.10 (d, 1H, *J* = 2.9 Hz), 6.91 (d, 1H, *J* = 8.9 Hz), 6.66 (dd, 1H, *J* = 8.9, 3.0 Hz), 5.92 (ddt, 1H, *J* = 16.9, 10.8, 5.5 Hz), 5.86 (d, 1H, *J* = 5.7 Hz), 5.75 (d, 1H, *J* = 5.5 Hz) 5.32 (d, 1H, *J* = 17.2 Hz), 5.25 (d, 1H, *J* = 10.4 Hz), 5.03 (bs, 1H), 4.65 (m, 1H), 4.59 (m, 1H), 3.14 (dd, 1H, *J* = 14.0, 5.9 Hz), 2.98 (d, 1H, *J* = 14.0, 6.5 Hz)

^{13}C NMR (122.3 MHz, $CDCl_3$) δ 170.0, 169.5, 155.5, 153.3, 150.6, 150.2, 141.2, 136.9, 132.4, 124.9, 118.5, 117.3, 115.3, 91.2, 85.5, 80.2, 66.3, 60.6, 36.5

HRMS [ESI(+)TOF]: calculated for $C_{22}H_{20}I_3NNaO_8$ $[M+Na]^+$ 829.8215; found 829.8208

Synthesis of caged coumarin 13 (for Supplementary Figure 8)

7-Amino-4-methylcoumarin **12** (300 mg, 1.71 mmol) was dissolved in dry pyridine (18 ml). After cooling on an ice bath, allylchloroformate (309.6 mg, 2.57 mmol, 1.5 eq.) was added under an N_2 atmosphere and the mixture was stirred for 20 h at ambient temperature. Pyridine was co-evaporated with toluene under reduced pressure. The evaporated mixture was dissolved in dichrolomethane and washed with HCl_{aq} (1 M) and $NaHCO_3$. The organic layer was dried with Na_2SO_4. The volatiles were evaporated under reduced pressure to yield 308 mg (70%) of the title compound **13** as a pale yellow solid.

^{1}H NMR (400.1 MHz, DMSO-d_6) δ 10.22 (s, 1H), 7.65 (d, 1H, J = 8.7 Hz), 7.52 (d, 1H, J = 2.1 Hz), 7.38 (d, 1H, J = 8.7, 2.1 Hz), 6.20 (d, 1H, J = 1.3 Hz), 5.99 (ddt, 1H, J = 17.3, 10.8, 5.5 Hz), 5.38 (dq, 1H, J = 17.3, 1.7 Hz), 5.26 (dq, 1H, J = 10.4, 1.5 Hz), 4.65 (dt, 1H, J = 5.55, 1.5 Hz), 2.36 (d, 1H, J = 1.3 Hz),

^{13}C NMR (100.6 MHz, DMSO-d_6) δ 160.0, 153.8, 153.1, 153.0, 142.7, 133.0, 125.9, 118.0, 114.3, 114.21, 111.89, 104.41, 65.1, 18.0

HRMS [ESI(+)TOF]: calculated for $C_{14}H_{14}NO_4$ $[M+H]^+$ 260.0917; found 260.0918

Does dependency of T_3 7 and AM-AT_3 8 on the gene switch (for Supplementary Figure 2)

The cell culture medium C of the 24 well plate, which is mentioned in the Methods section of cell culture and transfection in the main text, with the designer HEK-293T cells was replaced by media C containing various concentrations of T_3 **7** and AM-AT_3 **8** with 0.5 % DMSO. The cells were incubated at 37 °C in a humidified atmosphere containing 5% CO_2 for 24 h. Then, SEAP and sec-nluc activity was assayed as described in the Methods section of activity assays of SEAP and sec-nluc in the main text.

Determination of the appropriate concentration of AM-AT_3 8 (for Supplementary Figure 4)

Intracellular catalysis with $\mathbf{2}_1\mathbf{5}_2\subset$Sav (0.25 µM **2**, 0.5 µM CPD **5** and 0.25 µM Sav) was investigated using increasing concentrations AM-AT_3 **8**. The detailed experiment conditions are described in the Methods section of intracellular catalysis of ArM $\mathbf{2}_x\mathbf{5}_y\subset$Sav and FACS analysis in the main text.

Microscopy to confirm intracellular uptake of ArMs (for Supplementary Figure 5)

As described in the Methods section of cell culture and transfection in the main text, the HEK-293T cells transfected with pSP27, pYO1, and pSEAP2-control were prepared and pooled into a collagen-coated eight-well chambered slide with a two-times diluted concentration and cultivated. ArM $\mathbf{2}_1\mathbf{5}_2\subset$Sav and ArM $\mathbf{2}_1$(Atto565-Biotin)$_2\subset$Sav was diluted by a factor fifty with medium B. The culture medium C of the eight-well chambered slide with the designer HEK-293T cells was replaced by media B containing either ArM $\mathbf{2}_1\mathbf{5}_2\subset$Sav or ArM $\mathbf{2}_1$(Atto565-Biotin)$_2\subset$Sav. After 1 h incubation, cells were washed three times with medium B containing heparin (0.1 mg/ml). The presence of fluorescent compounds was analyzed using a confocal laser scanning microscope (Leica SP5) equipped with x63 oil immersion objective lens. An argon laser was used as light source (13% laser power) with excitation 561 nm and emission 571 ~ 650 nm (Leica HyDTM detector).

Supplementary References

1 Saxena, P., Hamri, G. C.-E., Folcher, M., Zulewski, H., Fussenegger, M. Synthetic gene network restoring endogenous pituitary-thyroid feedback control in experimental Graves' disease. *Proc. Natl. Acad. Sci. USA* **113**, 1244 (2016).

2 Kojima, R., Bojar, D., Rizzi, G., Hamri, G. C.-E., El-Baba, M. D., Saxena, P., Ausländer, S., Tan, K. R., Fussenegger, M. Designer exosomes produced by implanted cells intracerebrally deliver therapeutic cargo for Parkinson's disease treatment. Nat. Commun. **9** (2018), doi: http://10.1038/s41467-018-03733-8

3 Loosli, A., Rusbandi, U. E., Gradinaru, J., Bernauer, K., Schlaepfer C. W., Meyer, M., Mazurek, S., Novic, M., Ward, T. R. (Strept)avidin as host for biotinylated coordination complexes: stability, chiral discrimination, and cooperatively. *Inorg. Chem.* **45**, 660 (2006)

4 Gasparini, G. Matile, S. Protein delivery with cell-penetrating poly(disulfide)s. *Chem. Commun.* **51**, 17160 (2015).

5 Köhler, V., Mao, J., Heinisch, T., Pordea, A., Sardo, A., Wilson, Y. M., Knörr, L., Creus, M., Prost, J.-C., Schirmer, T., Ward, T. R. OsO_4.streptavidin: a tunable hybrid catalyst for the enantioselective cis-dihydroxylation of olefins. *Angew. Chem., Int. Ed.* **50**, 10863 (2011).

6 Mallin, H., Hestericová, M., Reuter, R., Ward, T. R. Library design and screening protocol for artificial metalloenzymes based on the biotin-streptavidin technology. *Nat. Protoc.* **11**, 835 (2016).

7 Derivery, E., Bartolami, E., Matile, S., Gonzalez-Gaitan, M. Efficient delivery of quantum dots into the cytosol of cells using cell-penetrating poly(disulfide)s. *J. Am. Chem. Soc.* **139**, 10172 (2017).

8 Völker, T. Dempwolff, F., Graumann, P. L., Meggers, E. Progress towards bioorthogonal catalysis with organometallic. *Angew. Chem., Int. Ed.* **53**, 10536 (2014).
